# Proton Exchange Membrane Water Splitting: Advances in Electrode Structure and Mass‐Charge Transport Optimization

**DOI:** 10.1002/adma.202416012

**Published:** 2025-03-04

**Authors:** Wenting Feng, Bin Chang, Yuanfu Ren, Debin Kong, Hua Bing Tao, Linjie Zhi, Mohd Adnan Khan, Rashed Aleisa, Magnus Rueping, Huabin Zhang

**Affiliations:** ^1^ Center for Renewable Energy and Storage Technologies (CREST) Physical Science and Engineering Division King Abdullah University of Science and Technology Thuwal 23955‐6900 Kingdom of Saudi Arabia; ^2^ KAUST Catalysis Center (KCC) Division of Physical Science and Engineering King Abdullah University of Science and Technology (KAUST) Thuwal 23955‐6900 Kingdom of Saudi Arabia; ^3^ School of Materials Science and Engineering Advanced Chemical Engineering and Energy Materials Research Center China University of Petroleum (East China) Qingdao 266580 P. R. China; ^4^ State Key Laboratory for Physical Chemistry of Solid Surfaces Collaborative Innovation Center of Chemistry for Energy Materials, and College of Chemistry and Chemical Engineering Xiamen University Xiamen 361005 P. R. China; ^5^ Fuels & Chemicals Division Research & Development Center, Saudi Aramco Dhahran 31311 Saudi Arabia; ^6^ Institute for Advanced Interdisciplinary Research (iAIR) School of Chemistry and Chemical Engineering University of Jinan Jinan 250022 P. R. China

**Keywords:** acidic oxygen evolution reaction, catalyst deactivation, membrane electrode assembly, proton exchange membrane water electrolyzers, stability optimization

## Abstract

Proton exchange membrane water electrolysis (PEMWE) represents a promising technology for renewable hydrogen production. However, the large‐scale commercialization of PEMWE faces challenges due to the need for acid oxygen evolution reaction (OER) catalysts with long‐term stability and corrosion‐resistant membrane electrode assemblies (MEA). This review thoroughly examines the deactivation mechanisms of acidic OER and crucial factors affecting assembly instability in complex reaction environments, including catalyst degradation, dynamic behavior at the MEA triple‐phase boundary, and equipment failures. Targeted solutions are proposed, including catalyst improvements, optimized MEA designs, and operational strategies. Finally, the review highlights perspectives on strict activity/stability evaluation standards, in situ/operando characteristics, and practical electrolyzer optimization. These insights emphasize the interrelationship between catalysts, MEAs, activity, and stability, offering new guidance for accelerating the commercialization of PEMWE catalysts and systems.

## Graphical abstract

This review probes recent advancements in PEMWEs for green hydrogen production from the perspective of acidic OER, identifies challenges related to corrosive environments and oxidative conditions, and proposes strategies to enhance the long‐term stability of PEMWEs by addressing both catalyst and membrane electrode assembly deactivation.

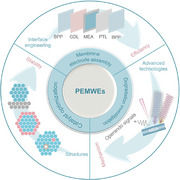

## 1 Introduction

**Figure 1:**
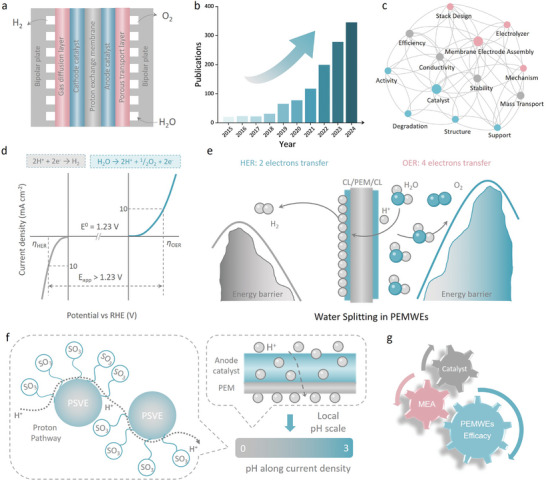
a) Schematic structure of PEM electrolyzer. b) Statistics of PEMWEs‐related publications in the past decade. Data from Google Scholar. c) Keywords related to PEMWEs research. d) Representative polarization curves of OER and HER. e) Schematic diagram of the difference in reaction barriers between HER and OER. f) Schematic diagram of the main structure of the proton exchange membrane and the local acidic environment. g) Synergistic relationship between catalyst, MEA, and operating efficiency of PEMWEs.

Hydrogen, produced with low greenhouse gas (GHG) emissions, is an attractive energy carrier for decarbonizing sectors such as heavy‐duty transport, industry (steel, chemicals, cement, etc.), power generation, and energy storage.^[^ ^1^ ^]^ Water electrolysis powered with renewable electricity has emerged as a key strategy for the production of low‐GHG hydrogen.^[^ ^2^ ^]^ The use of alkaline water electrolysis is attractive due to the low cost and stability of catalysts, but it faces technical bottlenecks such as high ohmic resistance, slow reaction kinetics, and low current density.^[^ ^3^ ^]^ These challenges limit its ability to meet the demands of future large‐scale, dynamic energy systems. In contrast, proton exchange membrane water electrolysis (PEMWE) presents a more promising route to industrial application, offering advantages such as compact system design, lower ohmic losses, higher current density, and superior gas purity (**Figure** **1**a).^[^ ^4^ ^]^ Over the past decade, with the growing demand for renewable energy‐driven green hydrogen production, research on PEMWE technology has increasingly gained attention (Figure 1b).^[^ ^5^ ^]^

The core advantage of PEMWE technology lies in its ability to provide rapid proton conductivity, reducing system ohmic resistance and enabling PEMWE to operate at high current densities exceeding 2 A cm^−2^ under a wide range of conditions.^[^ ^6^ ^]^ More importantly, PEMWE effectively integrates with intermittent renewable energy sources, such as wind and solar, offering superior dynamic response and a large‐scale energy storage solution.^[^ ^7^ ^]^ This capability could support large‐scale terawatt‐level renewable energy production and conversion. However, despite PEMWE's significant potential, the operation of anode catalysts under extreme conditions of high acidity (pH < 3) and oxidative environments leads to catalyst dissolution and electrode degradation, severely impacting long‐term operational efficiency. The issues of activity and stability remain major obstacles to widespread adoption (Figure 1c). These challenges hinder the commercialization of PEMWE and have compelled researchers to focus research efforts on optimizing catalysts, MEAs, and system designs to overcome the OER kinetic limitations while enhancing anode catalyst stability under harsh operating conditions (Figure 1d).^[^ ^8^ ^]^ Advancements in these areas are critical for the large‐scale deployment of PEMWE technology for sustainable hydrogen production.

OER, a key step in PEM water electrolysis, is a complex four‐proton‐electron coupled pathway, which results in slower reaction kinetics and serves as the bottleneck for overall water splitting.^[^ ^9^ ^]^ The kinetic limitations of OER not only require higher energy input but also impose stringent demands on catalyst design. In PEMWE, water is split at the anode to generate protons and electrons. The protons then travel through the proton exchange membrane to the cathode, where they recombine with electrons to form hydrogen (Figure 1e).^[^ ^10^ ^]^ and thus the efficiency of proton transport is crucial for the overall system performance. Proton exchange membranes are typically made of perfluorosulfonic acid (PFSA) fluoropolymers, which create a localized acidic environment. While these acidic conditions promote proton transport, it also exacerbate the degradation of both catalyst and membrane materials (Figure 1f).^[^ ^11^ ^]^ Under these conditions, the dissolution rate of the catalyst increases, and membrane electrode assemblies (MEA) become contaminated.^[^ ^12^ ^]^

By developing novel catalyst materials and optimizing MEA design, significant improvements have been achieved, impeding catalyst dissolution (Figure 1g). Furthermore, significant advancements have been made in enhancing the transport efficiency of electrons, protons, and gases at the MEA triple‐phase boundary through structural optimization, which is critical to practical industrial application. This review covers the critical topic of the anode electrode structure and mass‐charge transport optimization for PEMWE, to address the performance and long‐term stability of PEMWE. The review summarizes the potential deactivation mechanisms of PEMWE catalysts under acidic conditions and recent advances in catalyst design aimed at mitigating these challenges. The review also details crucial factors affecting assembly instability in complex reaction environments, including catalyst degradation and the dynamic behavior of the MEA triple‐phase boundary, resulting in cell failure. Targeted solutions on catalyst synthesis, MEA design, and operation strategies are proposed to reduce cost and improve the long‐term stability and performance of PEMWE. Finally, the review article provides detailed perspectives on the importance of strict activity/stability evaluation standards, in situ/operando characterization, and practical electrolyzer optimization. These insights emphasize the interrelationship between catalysts, MEAs, activity, and stability, offering new guidance for accelerating the commercialization of PEMWE systems.

## 2 PEMWEs Deactivation Mechanisms

Understanding the deactivation mechanisms in PEMWEs is critical to enhance durability and optimize performance. Catalyst deactivation, one of the main challenges, arises from issues such as structural reconstruction, overoxidation, and dissolution all of which severely impair performance. MEA deactivation is linked to the parallel degradation of the proton exchange membrane and anode electrode in acidic environments, leading to reduced ionic conductivity and compromised mechanical integrity.^[^ ^9^, ^12^ ^]^ Additionally, device shutdown involves broader systemic issues, including the accumulation of contaminants, gases, and other operational strategies.^[^ ^13^ ^]^ This section explores the key factors impacting stability and proposes solutions to mitigate deactivation mechanisms, ultimately enhancing the performance and reliability of PEMWEs in acidic OER applications.

### 2.1 Catalyst Deactivation

#### 2.1.1 Surface Passivation

**Figure 2:**
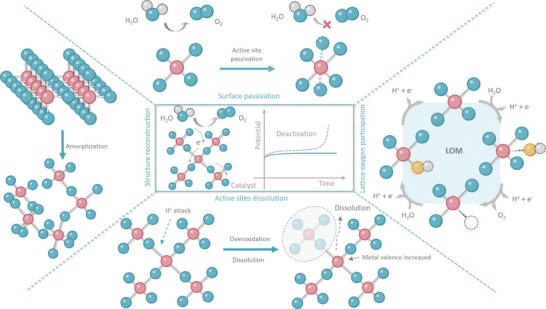
Schematic diagram of catalyst deactivation mechanism including a) catalyst surface passivation, b) lattice oxygen participation, c) active sites dissolution, and d) structure reconstruction.

Surface passivation refers to the formation of an inactive or insulating layer on the catalyst surface, which inhibits access to active catalytic sites and impairs the overall reaction efficiency (**Figure** **2**a).^[^ ^14^ ^]^ The OER in PEMWEs occurs at high potentials and involves the transfer of four protons and four electrons per oxygen molecule produced. Under these extreme conditions, the catalyst, typically composed of precious metals like iridium or ruthenium, becomes prone to oxidation. For example, in iridium‐based catalysts, iridium oxides (IrO_x_) form on the catalyst surface during extended operation. While IrO_x_ is initially active for the OER, prolonged exposure leads to the formation of more stable but less catalytically active phases, which cover the active sites necessary for OER, resulting in surface passivation.^[^ ^15^ ^]^ Passivation occurs when a protective oxide or hydroxide layer forms on the catalyst surface, effectively insulating the underlying active material from the reactants. This barrier restricts the access of water molecules to the active sites and obstructs the transfer of electrons and protons, which are essential for the OER. The degree of passivation depends on the nature of the catalyst material, the operational environment, and the duration of the electrolysis process. During prolonged OER operation, particularly at high anodic potentials, these oxide films grow on the catalyst surface, reducing the availability of iridium active sites for oxygen evolution. The insulating properties of the IrO_x_ layer obstruct proton transport and impede electron transfer, slowing down the reaction kinetics and ultimately decreasing the overall efficiency of the PEMWE system.

#### 2.1.2 Lattice Oxygen Participation

The stability of catalysts during the OER is also significantly influenced by the pathways involved in the reaction. The adsorbate evolution mechanism (AEM) primarily describes the relationship between the catalyst's structure and its activity. In contrast, lattice oxygen‐mediated mechanism (LOM) is concerned with the origin and development of oxygen intermediates during the OER process. When the energy level of the oxygen p‐band center is relatively low, the lattice oxygen tends to be more confined, and the metal oxide typically adheres to the AEM mechanism. Conversely, when the oxygen p‐band center energy level is higher, the lattice oxygen exhibits greater freedom, leading the metal oxide to follow the LOM mechanism.^[^ ^16^ ^]^ Therefore, the LOM can enhance OER activity but may also induce lattice structural instability, contrasting with the catalytically stable yet less active AEM pathway.

In the LOM pathway, lattice oxygen participation also requires the replenishment of remaining oxygen vacancies to sustain the reaction (Figure 2b).^[^ ^17^ ^]^ However, the rate of oxygen vacancy refilling is typically slower than the generation rate of oxygen vacancy defects in LOM. This imbalance leads to the etching out of adjacent cations from the catalyst surface to maintain charge equilibrium. Additionally, dissolved metal cations may diffuse away from the electrode and undergo oxidation to higher valence states with increased solubility in the electrolyte. LOM predominantly occurs in materials like RuO_2_, perovskites, and pyrochlores due to their redox flexibility and structural tolerance.^[^ ^18^ ^]^ In contrast, rutile IrO_2_ exhibits stronger Ir─O bonding, making LOM less prevalent in this structure. Therefore, the dominant OER mechanism of specific catalysts is achieved through careful composition and structural design. Chung et al. investigated various A_x_Ir_y_O_z_‐type oxides (where A = Ca, Sr, Ba, Y, Pr, and Nd), providing insights into the structure‐property relationships across these materials.^[^ ^19^ ^]^ Strong connectivity between [IrO_6_] octahedra in configurations involving closed‐edge or face‐sharing arrangements significantly impacts the development of efficient and stable OER catalysts under harsh acidic conditions. This study underscores the importance of understanding structure‐property correlations in A_x_Ir_y_O_z_‐type oxides, suggesting potential for discovering new iridates with robust connectivity. Adjusting the active site configuration and coordination environment offers prospects for optimizing stability during the LOM process. However, achieving the ability to switch between AEM and LOM pathways selectively in a catalyst, while maintaining constant chemical components, remains a challenging yet intriguing goal.

#### 2.1.3 Active Sites Dissolution

A significant aspect of catalyst deactivation involves active‐site dissolution (Figure 2c). Due to the harsh conditions of OER in acidic media, only a select few catalysts maintain stability during oxidation. The dissolution process under these oxidative environments during electrochemical reactions is quite intricate.^[^ ^20^ ^]^ In addition to the inherent stability of catalysts, research indicates that various catalytic facets, support substrates, and reaction potentials can influence distinct dissolution pathways. Moreover, dissolved catalysts or ions in the electrolyte may impact catalytic performance or undergo redeposition processes during electrochemical reactions. Therefore, further studies are essential to elucidate the intricate mechanisms governing catalyst dissolution.

Catalyst dissolution in OER environments is primarily influenced by the intrinsic elemental properties of the catalysts.^[^ ^21^ ^]^ The Pourbaix diagram serves as a critical tool for evaluating the thermodynamic stability of various metals under specific acidic conditions, illustrating their stability across different redox states, pH levels, and applied voltages. For instance, IrO_2_ is renowned for its stability in acidic media, maintaining robust performance except at extremely high voltages, where it may become susceptible to dissolution.^[^ ^22^ ^]^ This is due to the strong Ir─O bonds that confer stability but may become weakened under extreme conditions, leading to potential dissolution. In contrast, RuO_2_, while initially promising, demonstrates significant instability under acidic conditions at elevated potentials. This instability is attributed to overoxidation, where RuO_2_ transforms into soluble RuO_4_, leading to a loss of catalytic material and performance degradation.^[^ ^23^ ^]^ Transition metal‐based materials, such as spinel Co_3_O_4_, also face substantial challenges. Although these materials show theoretical activity that rivals benchmark catalysts like RuO_2_, their stability in acidic environments remains a significant hurdle. The instability of Co_3_O_4_ in acidic conditions often results in degradation and dissolution, undermining its potential as an effective OER catalyst.^[^ ^24^ ^]^ These observations underscore the necessity of carefully selecting and engineering catalyst materials to ensure both high activity and stability under acidic OER conditions. Understanding these dissolution mechanisms through tools like the Pourbaix diagram is crucial for developing more durable and effective catalysts for industrial applications.

#### 2.1.4 Structure Reconstruction

Structural reconstruction refers to the significant changes in the physical and chemical structure of a catalyst during OER operation, which can drastically affect both its activity and long‐term stability. This phenomenon is often driven by the harsh operating conditions present at the anode in PEMWEs, where high anodic potentials, combined with an aggressive acidic and oxidative environment, exert substantial stress on the catalyst material. These conditions can trigger a range of structural alterations, including phase transitions, morphological changes, surface restructuring, and even complete catalyst dissolution.^[^ ^25^ ^]^

One common manifestation of structural reconstruction is phase transition, wherein the catalyst changes from one crystalline phase to another under OER operating conditions (Figure 2d). For instance, many transition metal oxides and perovskite catalysts are prone to phase segregation, where the material separates into distinct phases, each with different chemical and physical properties.^[^ ^26^ ^]^ This can lead to the formation of new crystalline phases that are less catalytically active than the original structure, thereby reducing the availability of active sites necessary for efficient oxygen evolution. Such transformations disrupt the coordination environment of the active sites, lowering the intrinsic catalytic activity and overall system efficiency. Moreover, the highly dynamic environment of OER can induce significant changes in catalyst morphology. Prolonged exposure to high potentials and acidic electrolytes often leads to the dissolution of surface atoms, resulting in the roughening or restructuring of the catalyst surface. As the surface becomes increasingly disordered, active sites may be lost, and less favorable surface facets, which are less conducive to OER, may dominate. This structural degradation is particularly detrimental for nanoparticle catalysts, where a significant portion of catalytic activity is derived from surface atoms. In more severe cases, structural reconstruction can lead to the formation of cracks, voids, or pores within the catalyst.^[^ ^27^ ^]^ These structural defects not only reduce the mechanical stability of the catalyst but also increase its exposure to the electrolyte, accelerating further degradation. Cracks and voids can act as channels through which the acidic electrolyte penetrates deep into the catalyst, exacerbating the dissolution and leaching of active materials. This self‐reinforcing degradation mechanism results in a continuous loss of catalytic material, which compromises both the activity and durability of the system.

### 2.2 Deactivation of MEA

**Figure 3:**
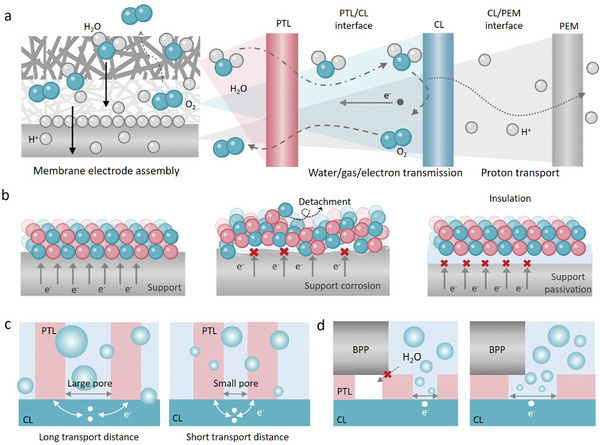
a) Schematic diagram of membrane electrode components and the transport of water, gas, protons, and electrons in the membrane electrode. b) Schematic diagram of electrode failure mode and slow electron transport. Support deactivation mechanism of dissolution corrosion and passivation. c) Schematic diagram of electron transmission distance in different pores of PTL. d) Schematic of the effect of different pore states of PTL and BPP on the catalytic process.

In a PEMWE, MEA is central to the electrolysis process, integrating several essential functions into a single, cohesive unit (**Figure** **3**a). The MEA consists of three primary components: the catalyst layer (CL), the proton exchange membrane (PEM), and the porous transport layer (PTL).^[^ ^12a^ ^]^ The catalyst layer, which is bonded to the PEM, drives the electrochemical reactions required for oxygen production.^[^ ^28^ ^]^ The PEM serves as the crucial conduit for proton transport, facilitating the movement of protons from the anode to the cathode while preventing the migration of gases and electrons. Meanwhile, the PTL provides pathways for gas bubbles, formed during the OER, to escape without hindering the access of reactants to the catalyst sites. Efficient charge transport is essential for maintaining high current densities and minimizing resistive losses, as any disruption in the transport of protons or electrons can lead to decreased reaction rates and reduced system efficiency. Furthermore, effective management of gas bubbles can prevent blockage of active sites, ensuring sustained catalytic activity and overall system durability. Thus, understanding and optimizing these transport phenomena are critical for enhancing the efficiency and longevity of PEMWE technology in sustainable hydrogen production.

#### 2.2.1 Electrode Corrosion

In applications involving acidic electrolytes, reducing the cost of precious‐metal oxides used as OER catalysts is crucial for enhancing their feasibility and efficiency. A practical approach to achieving this involves depositing IrO_2_‐based and RuO_2_‐based catalysts onto stable and conductive support materials (Figure 3b). This method addresses several key issues including helping prevent catalyst aggregation, improving electrical conductivity, and maximizing the effective catalytic surface area exposed to the electrolyte.^[^ ^29^ ^]^ Each of these factors plays a significant role in enhancing the mass activities of precious‐metal oxide OER catalysts, ultimately contributing to more effective and economical catalytic processes. The performance of supported electrocatalysts is significantly influenced by both the conductive substrate and the interlayer between the substrate and the electrocatalysts, a phenomenon known as the substrate effect.^[^ ^30^ ^]^ This substrate effect is critical in determining the surface properties, electrocatalytic stability, and overall activity of the catalysts. The quality of the conductive substrate impacts how well it can conduct electricity and support the catalyst layer, while the interlayer's properties can affect the interaction between the catalyst and the substrate. Therefore, understanding and optimizing these interactions are essential for developing efficient and durable OER catalysts that can withstand the demanding conditions of acidic environments.

Unfortunately, conventional support materials, such as carbon, transition metal sulfides, nitrides, and phosphides, often encounter significant issues in acidic and oxidative environments. These materials are prone to corrosion, a process where the support material gradually deteriorates due to chemical reactions with its environment. In acidic conditions, the support materials can undergo oxidative dissolution, leading to the release of metal ions into the electrolyte and a subsequent loss of structural integrity.^[^ ^31^ ^]^ For instance, carbon materials, when exposed to high oxidative potentials, can be oxidized to CO_2_, which not only results in the loss of the support material but also reduces the number of conductive pathways linking the catalyst to the electrode.^[^ ^32^ ^]^ As the support material corrodes, its ability to maintain electrical conductivity and provide a stable platform for the catalyst diminishes. This degradation directly impacts the performance of the electrode, as the reduced connectivity between the catalyst and the electrode leads to poor electrical contact and less efficient catalysis. Moreover, the physical degradation of the support material can cause additional issues, such as the detachment of active catalyst particles from the support, further diminishing the catalyst's effectiveness. Borup et al. observed the inevitable thinning of catalyst electrodes over prolonged operation, as evidenced by cross‐sectional SEM analysis.^[^ ^33^ ^]^ The primary cause of this thinning was attributed to the support material, underscoring the importance of selecting corrosion‐resistant supports to mitigate electrode detachment.

In addition, while metal substrates with high conductivity, such as titanium, generally exhibit good stability, they often face challenges related to surface passivation.^[^ ^31^ ^]^ Passivation refers to the process by which a material becomes coated with a protective layer that prevents further chemical reactions. While passivation can be beneficial in some contexts, in the case of support materials for OER catalysts, it often leads to undesirable outcomes. In acidic environments, passivation layers can form on the support materials, which may initially protect against further corrosion but can also interfere with the interaction between the catalyst and the substrate. For example, passivation layers can reduce the surface area available for catalytic reactions by covering active sites on the support material. This reduction in available active surface area can impede the catalyst's performance by limiting the number of active sites that participate in the OER process. Additionally, passivation layers can alter the electronic properties of the support, affecting its ability to conduct electricity effectively.^[^ ^34^ ^]^ This change in electronic properties can further degrade the overall catalytic activity and stability of the system. The combined effects of corrosion and passivation significantly contribute to the decline in catalytic performance over time. The deterioration of the support material not only affects the stability and activity of the catalyst but also promotes phenomena such as particle migration, coalescence, and agglomeration. These issues can lead to the detachment of catalyst particles, reducing the effective catalytic surface area and further diminishing performance.

To address these challenges, advanced material design and engineering strategies are crucial. Researchers are exploring the development of more resilient and corrosion‐resistant support materials, as well as innovative coating and modification techniques to enhance the durability and stability of the supports. By improving the resistance of support materials to corrosion and passivation, it is possible to advance the development of cost‐effective and durable precious‐metal oxide catalysts, tailored specifically for use in acidic electrochemical environments. Such advancements are vital for achieving long‐term performance and reliability in practical applications of OER catalysts.

On the other hand, PTL is a vital multifunctional layer sandwiched between CL and BPP. The structure of PTL, especially porous structure is crucial to determine charge, mass, and heat transport properties. When the pores of the PTL are excessively large, this leads to an increase in the distance over which electrons must travel (Figure 3c).^[^ ^35^ ^]^ On the other hand, if the PTL pores are too small, it can obstruct the contact between the flow field and the catalyst. As depicted in Figure 3d, if the catalyst surface is entirely covered due to inadequate pore size, the catalyst becomes inaccessible for reaction, impeding the overall electrochemical process.^[^ ^36^ ^]^ Even when the coverage is slightly reduced, and some reaction begins, the excessively small pores can hinder the effective flow of reactants and block the overflow of bubbles, thereby disrupting the reaction dynamics. This increased transmission distance results in higher ohmic impedance, which can reduce the overall efficiency of the electrolyzer. Therefore, both excessively small and excessively large pores in the PTL can negatively impact the performance of PEMWEs. Proper PTL design is critical to ensure adequate contact between the flow field and catalyst while optimizing pore size to balance efficient electron transport and effective gas management. In addition, BBPs play a crucial role in the membrane electrode assembly of PEMWEs. They are responsible for conducting electrons, regulating flow fields, and managing heat diffusion. To ensure efficient current distribution and heat management, bipolar plates must have high electrical and thermal conductivity. Poor conductivity can lead to increased resistance and reduced efficiency. Over time, however, bipolar plates may suffer from corrosion or erosion, particularly if they are not made from materials resistant to the acidic or basic environment within the electrolyzer. This degradation can further increase resistance and decrease efficiency. Additionally, inadequate design or blockage of gas flow channels in the bipolar plates can hinder effective gas removal, further compromising the overall performance of the electrolyzer. In summary, maintaining the integrity and optimal design of bipolar plates is essential for the efficient operation of PEM electrolyzers. At the same time, the BBP design must be carefully adjusted and optimized based on the specific operating conditions of the PEMWEs.

#### 2.2.2 Bubble Effect

**Figure 4:**
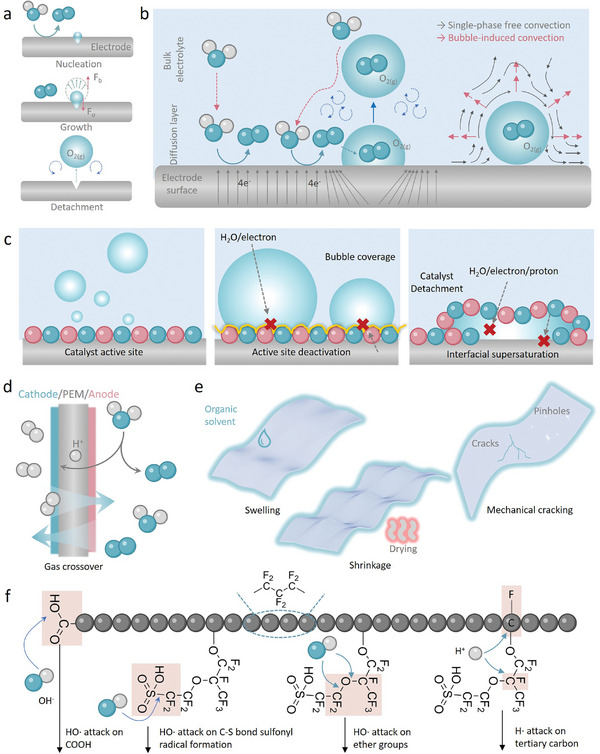
a) The various stages of O_2_ bubble evolution. b) Transport and electrochemical processes in gas‐evolving electrodes. The schematics of bubble issues in MEA. c) Schematic diagram of the effect of gas accumulation on catalyst, bubble coverage, and interfacial supersaturation. d) Schematic diagram of O_2_ and H_2_ crossover in membrane electrode. e) Physical failure of proton exchange membranes, swelling, shrinkage, and stress‐induced cracking. f) Chemical failure of proton exchange membranes. Summary of the chemical degradation mechanisms of radical attack on the Nafion polymer structure.

Layer detachment and blockage of active sites due to bubble release have been identified as significant factors contributing to stability loss. The formation of bubbles during OER is a common occurrence attributable to the limited solubility of molecular oxygen in the electrolyte. At the heterogeneous interface, bubbles undergo nucleation, growth, and detachment, impacting the mechanical stability of the catalyst on the electrode (**Figure** **4**a).^[^ ^37^ ^]^ When bubbles grow or detach, electrolytes can fill the pores of the catalyst, deactivating heterogeneous nucleation sites. Furthermore, gas bubbles generated during OER at high current densities exhibit distinct physical properties compared to those in laboratory‐scale tests. At low current densities, the electrode operates primarily in a single‐phase regime where the reaction proceeds uniformly across the anode surface. The rate of oxygen formation is sufficiently low to allow for its diffusion into the electrolyte before reaching the nucleation threshold for bubble formation. This scenario typically involves mild conditions and results in minimal damage to the electrodes. As current density increases, bubbles begin to form on the electrode surface via heterogeneous nucleation and grow until they reach a detachment threshold. During bubble growth, the effective electrocatalytic area is reduced, leading to a non‐uniform distribution of current density (Figure 4b). This compression of current lines near the catalyst‐electrolyte interface adjacent to the bubbles results in localized increases in current density. In these high‐density regions, reactant depletion accelerates the rate of oxygen generation, further promoting bubble growth. The continuous growth and detachment of bubbles under these conditions generate significant mechanical stress, ultimately leading to catalyst detachment (Figure 4c).^[^ ^38^ ^]^ Furthermore, the study suggests that for general OER catalysts, including commercially available random nanoparticles and 3D‐nanostructured catalysts, multiple factors influence bubble dynamics beyond mere detachment from catalytic surfaces. These factors include bubble coalescence and removal from the catalyst layer.^[^ ^39^ ^]^ To explore these mechanisms, two hypotheses were formulated based on electrochemical analysis data: i) the open and organized 3D structure reduces the residence time of bubbles within the catalyst layer, and ii) prolonged residence time increases the likelihood of bubble coalescence in the fluid.^[^ ^40^ ^]^ Based on the above perspective, Jung et al. developed a woodpile structure catalyst using 3D stack printing to enhance bubble transport and minimize surface charge accumulation, thereby ensuring both catalyst activity and stability. Therefore, in the future design of catalyst microstructures, the catalysts with unique 3D structures can promote the rapid transport of bubbles as well as detachment, thus avoiding the mechanical stress generated by bubbles to cause electrode detachment and achieving stable catalytic performance.

#### 2.2.3 Proton Exchange Membrane Decay

The degradation of the PEM in the PMEWEs is a critical concern, as it plays a vital role in ensuring the efficient operation of proton transport. When the membrane deteriorates, it can lead to reduced adhesion of the catalyst, causing it to delaminate and impairing proton transport. This attenuation can significantly hinder the electrolysis process and create conditions for gas crossover, where hydrogen and oxygen gases can mix (Figure 4d). This is particularly hazardous, as the presence of hydrogen in the air can create explosive mixtures; the lower explosive limit for hydrogen is ≈4% by volume. Therefore, even a minor breach in the integrity of the PEM can pose serious safety risks, potentially leading to explosive events under certain operational conditions. Consequently, maintaining the structural and functional integrity of the PEM is paramount for ensuring both the efficiency and safety of PEMWE systems.

Physical or mechanical failures are associated with structural and physical changes in the MEA components due to processing conditions and operational stresses. One prevalent issue is swelling and shrinkage (Figure 4e), which usually occurs when the PEM interacts with solvents during catalyst loading or other operational stages, especially those that are polar or possess similar chemical properties to the membrane's polymer matrix.^[^ ^41^ ^]^ These solvents are absorbed by the membrane material, causing it to expand and increase in volume. The absorption of solvents leads to a significant change in the membrane's dimensions and mechanical properties, resulting in noticeable swelling and deformation. Shrinkage is more caused by the further process of solvent drying. During the solvent drying phase, the membrane undergoes a significant reduction in volume as the solvent evaporates. Initially, the PEM absorbs the solvent, causing it to swell and expand. However, as the drying process progresses, the removal of the solvent from the membrane results in a dramatic contraction. This shrinkage is primarily due to the loss of the absorbed solvent, which causes the membrane material to return to or even contract beyond its original dimensions. The consequences of swelling and shrinkage are multifaceted. As the membrane swells and shrinkage, it can experience delamination or a loss of adhesion with the catalyst layer. This loss of contact between the membrane and the catalyst can severely impact the efficiency of the electrochemical reactions. Delamination disrupts the integrity of the MEA, leading to reduced proton transport efficiency and compromised overall cell performance. Additionally, the mechanical deformation of the membrane can cause misalignment of the catalytic components, further diminishing the effectiveness of the electrolyzer.

Mechanical cracking and stress‐induced fractures are common issues in MEA, often resulting from various operational stresses and environmental conditions.^[^ ^41^ ^]^ Repeated heating and cooling cycles induce thermal stress, causing differential thermal expansion and contraction between the membrane and surrounding components, which can lead to cracks and fractures. Additionally, physical forces from pressure changes or variations in thermal expansion contribute to membrane cracking over time. Compression damage also poses a significant risk, as excessive or uneven compression during assembly can create localized stress concentrations, leading to deformation or cracking of the membrane. Furthermore, improper assembly practices, including misalignment or excessive compression, can exacerbate mechanical damage and reduce performance. Addressing these challenges involves optimizing operational conditions, carefully managing thermal and pressure variations, and ensuring precise assembly techniques to enhance the stability and performance of PEMWE systems. The distribution of stress, a crucial mechanical factor in electrolyzer assembly, significantly influences overall efficiency and stability. Traditional cell structures typically employ serpentine flow channels (S‐FC) to deliver reactants and manage product distribution, but they often result in uneven stress distribution. This uneven distribution places high stress on certain regions of the anode catalyst layer (ACL), causing severe deformation, while other regions experience lower activity due to poor electrical contact. Tao et al. proposed a solution with a Ti mesh flow channel (TM‐FC) featuring gradient pores to mitigate stress inhomogeneity.^[^ ^42^ ^]^ The ACL‐TM‐FC configuration exhibited superior performance both before and after durability testing. The adoption of TM‐FC not only addresses stress distribution issues but also enhances overall electrolyzer performance by ensuring stable operation and prolonged durability. This approach highlights the importance of mechanical design considerations in optimizing the efficiency and longevity of electrolyzer systems.

Chemical failures primarily involve degradation processes that occur within the harsh electrochemical environment of the electrolyzer. One of the major mechanisms of chemical failure is free radical attack (Figure 4f).^[^ ^43^ ^]^ During electrochemical reactions, highly reactive free radicals are generated, which can aggressively interact with the membrane and catalyst materials. These radicals initiate oxidative degradation, leading to the chemical breakdown of critical components such as the PEM and catalyst layers.^[^ ^44^ ^]^ This oxidative damage impairs the structural integrity and functional properties of the MEA, reducing its effectiveness and potentially leading to the failure of the electrolyzer. Further insights into the mechanisms of degradation in catalyst‐coated membranes of PEMWEs, particularly after extended operational periods, have been obtained through advanced analytical techniques such as electron microscopy and chemical analysis.^[^ ^45^ ^]^ These methodologies provide detailed imaging and compositional data, allowing researchers to observe the morphological changes and identify the specific chemical alterations that occur within the MEA. Such analyses have revealed the extent of damage inflicted by free radical attacks and other degradation processes, offering valuable information for developing strategies to enhance the durability and performance of PEMWE components. Understanding these degradation mechanisms is crucial for designing more robust materials and improving the longevity and reliability of PEMWEs in practical applications.

Another significant form of chemical failure in MEA is metal poisoning, which involves the contamination of the membrane by metal ions such as calcium, titanium, iron, and copper.^[^ ^46^ ^]^ These metal ions can originate from impurities in the feed water or from corrosion within the MEA, and they can cause substantial disruptions to the electrochemical process. The process of metal poisoning begins when these metallic ions dissolve into the membrane through mechanisms of diffusion and permeation. Once inside the PEM, these metal ions interact with and occupy the ion exchange sites, which are normally reserved for protons. The Nafion membrane, a commonly used proton exchange membrane, has a higher affinity for metal ions compared to protons. As a result, the mobility of the metal ions within the Nafion membrane is significantly lower than that of protons. This disparity in ion mobility leads to increased ohmic resistance, as the metal ions impede the efficient flow of protons through the membrane. The presence of these contaminants thus results in higher ohmic losses, which degrade the overall performance of the PEMWE. These higher ohmic losses manifest as reduced proton conductivity and impaired electrochemical efficiency, ultimately leading to diminished cell performance and potential operational issues. Furthermore, prolonged exposure to metal contaminants can exacerbate these issues, potentially leading to substantial operational failures and reduced system longevity. Therefore, addressing metal poisoning is crucial for ensuring the sustained performance of PEMWEs. This involves not only the development of more resilient catalyst materials but also the implementation of effective purification and maintenance strategies to minimize the risk of metal contamination. Both free radical attacks and metal poisoning underscore the necessity for robust material design and comprehensive mitigation strategies to maintain the stability and efficiency of the membrane electrode assembly in PEMWE systems.

## 3 Strategies to Improve Acidic OER Efficiency of Catalysts

Optimizing catalyst activity is critical for enhancing the efficiency of PEM electrolyzers. In PEM water electrolysis, OER is the rate‐limiting step, requiring a significant overpotential to proceed. Improving OER catalytic activity can significantly reduce the required overpotential, thereby lowering the energy consumption of water splitting and improving energy efficiency. Additionally, catalysts with higher activity and durability extend the lifespan of the electrolyzer, reduce maintenance and replacement costs, and increase the economic viability of large‐scale hydrogen production, supporting the transition to a more sustainable energy system. Thus, optimizing the activity and stability of catalysts is essential for improving the energy efficiency, reliability, and cost‐effectiveness of PEM electrolyzers, making them key to the future of clean hydrogen production.

### 3.1 Catalyst Microstructure Regulation

The performance of catalysts depends significantly on their structure, which governs both activity and stability. However, achieving a balance between high activity and robust stability remains a challenging task, as illustrated by commercial IrO_2_. IrO_2_ in its crystalline rutile phase demonstrates excellent structural stability but lacks the desired OER activity. In contrast, amorphous IrO_x_ exhibits superior activity but often suffers from compromised stability. The quest for catalysts with optimal activity‐stability balance drives continuous research in catalyst structure optimization. The structural characteristics of catalyst materials, including their morphology and atomic arrangements, play crucial roles in determining their electrocatalytic performances.^[^ ^47^ ^]^ Optimizing catalyst structure involves intrinsic strategies such as refining microstructural and electronic properties aimed at enhancing the stability of active sites within the catalytic environment.^[^ ^48^ ^]^ Strategies such as vacancy, metal doping, strain engineering, reconstruction, solid solution oxide formation, heterostructure creation, and composite material design have played crucial roles in improving stability while maintaining or enhancing catalytic performance.^[^ ^47^, ^49^ ^]^ These approaches harness the unique electronic and structural effects introduced by additional elements or carriers, thereby optimizing catalytic sites for stability in harsh acidic OER environments. Among these strategies, the atomic arrangement has garnered significant research attention. Manipulating atomic arrangement involves adjusting morphology, orientation, and degree of order, offering practical pathways to tailor the properties of catalysts. These adjustments influence the active sites' accessibility and interaction with reactants, ultimately impacting the efficiency and durability of electrocatalysts during OER.

**Figure 5:**
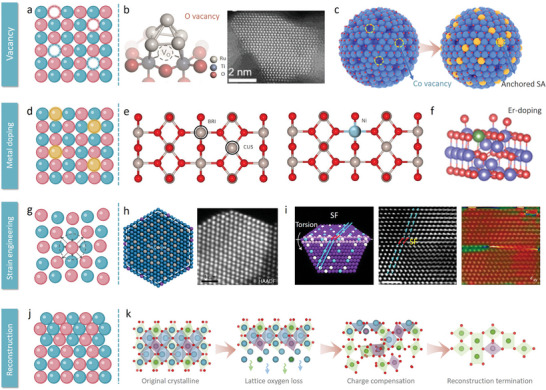
a) Schematic diagram of vacancy engineering. b) Atomic structure of Ru_5_ cluster adsorbed on the TiO_2_ (110) surface with single oxygen vacancy (V_O_‐Ru_5_/TiO_x_). Reproduced with permission.^[^ ^52^ ^]^ Copyright Year, Wiley. c) Schematic illustration of Co vacancy and a perfect surface for Ru anchoring. Reproduced with permission.^[^ ^53^ ^]^ Copyright 2023, American Chemical Society. d) Schematic diagram of metal doping. e) Atomistic structures of the surfaces of Ni‐RuO_2_ (110). Reproduced with permission.^[^ ^26b^ ^]^ Copyright 2023, Springer Nature. f) Schematic illustration of identified surface coverage of Er‐doped Co_3_O_4_ (311). Reproduced with permission.^[^ ^55^ ^]^ Copyright 2024, American Chemical Society. g) Schematic diagram of strain engineering. h) Atomic model and lattice resolved HRTEM image of Ru_1_‐Pt_3_Cu. Reproduced with permission.^[^ ^61^ ^]^ Copyright 2019, Springer Nature. i) Simulated structure of Ta_0.1_Tm_0.1_Ir_0.8_O_2‐δ_ with grain boundaries and corresponding lattice resolved HRTEM image and strain analysis. Reproduced with permission.^[^ ^62^ ^]^ Copyright 2021, Springer Nature. j) Schematic diagram of reconstruction. k) Surface reconstruction in Mo‐Pr_3_IrO_7_ during electrochemical reactions. Reproduced with permission.^[^ ^71^ ^]^ Copyright 2023, Wiley.

A notable advancement lies in ruthenium‐based materials with vacancy tuning (**Figure** **5**a). Despite exhibiting superior OER activity compared to IrO_2_, the stability of RuO_2_ remains a significant challenge.^[^ ^50^ ^]^ Structural collapse and dissolution under high potentials due to over‐oxidation of Ru (such as RuO_4_) are critical issues addressed through approaches like defect engineering, doping, strain effects, and structural tuning.^[^ ^51^ ^]^ These strategies aim to stabilize RuO_2_ by optimizing its electronic structure, thus preventing active site degradation, and enhancing overall durability in acidic environments. For example, Lv et al. introduced oxygen vacancies (V_O_) into RuO_2_, enhancing the adsorption strength of Ru_5_ clusters and stabilizing their structure against oxidation, thereby enhancing their antioxidative capability (Figure 5b).^[^ ^52^ ^]^ Simultaneously, Peng et al. pioneered an alternative approach by utilizing cobalt metal defect anchor sites on the support to precisely control the spatial positioning of Ru single atoms on the Co_3_O_4_ surface, thereby enhancing acidic OER activity (Figure 5c).^[^ ^53^ ^]^ The refined structural design enabled a shift in the reaction mechanism from the lattice oxygen mechanism (LOM) to an optimized adsorbate evolution mechanism (AEM). Notably, the Ru atoms embedded into cation vacancies revealed an optimized proton donor‐acceptor mechanism (PDAM), presenting a novel single‐atom catalytic pathway to bypass the traditional scaling relationship. At the anchored Ru─O─Co interface, spatial steric interactions with intermediates played a crucial role in optimizing intermediate configurations and lowering energy barriers. This strategy of anchoring active single atoms to metal defect sites offers a new catalytic approach that circumvents classical scaling relations.

Structural optimization based on metal‐doping remains crucial for the precise tuning of orbital filling and charge distribution of active elements within catalyst materials (Figure 5d). Wang et al. reported on Ni‐RuO_2_, where the introduction of nickel into the RuO_2_ lattice enhances the stability of surface Ru and subsurface oxygen by substituting fully coordinated bridge Ru sites (Figure 5e).^[^ ^26d^ ^]^ The incorporation of Ni into the lattice would increase the energy cost for Ru demetallation from 1.87 to 2.22 eV, clearly suggesting that the introduction of Ni dopants to the RuO_2_ lattice had also stabilized lattice oxygen during OER. This adjustment shifts the reaction pathway toward an AEM mechanism, thereby mitigating LOM and preventing Ru over‐oxidation.^[^ ^54^ ^]^ The catalyst demonstrated stability under water electrolysis conditions, providing at least 1000 h of operational stability at current densities up to 200 mA cm^−2^ in PEMWEs. Furthermore, incorporating stable elements through alloying or doping within catalysts alters electronic structures and chemical reactivity, aiming to strengthen bonding with electrode substrates and suppress dissolution. Recent studies highlight enhancements in durability and sustained catalytic activity in acidic media through strategies such as alloying iridium catalysts with tungsten or nickel. Techniques involving electrochemical ion insertion, and altering electronic or crystal structures through the introduction of foreign elements, are effective synthesis strategies to enhance catalytic performance. Zhang et al. introduced non‐stoichiometric Li into RuO_2_ via electrochemical methods, with Li embedded into the lattice gaps of RuO_2_ to provide electrons and distort the local structure.^[^ ^49d^ ^]^ Compared with the pristine RuO_2_, the intercalation of lithium generates more intense tensile‐compressing dislocation dipoles. Meanwhile, the inherent lattice strain results in the surface structural distortion of Li_x_RuO_2_ and activates the dangling O atom near the Ru active site as a proton acceptor, which stabilizes the OOH* and dramatically enhances the activity. More importantly, the Ru valence state is lowered with the formation of a stable Li‐O─Ru local structure, and the Ru─O covalency is weakened, which suppresses the dissolution of Ru, resulting in greatly enhanced durability. A similar strategy has been applied to non‐noble metal systems. Pan et al. proposed a method of enhancing the intrinsic OER activity and stability of Co_3_O_4_ through synergistic catalytic enhancement via Er doping (Figure 5f).^[^ ^55^ ^]^ Doping Co_3_O_4_ with Er induces structural defects and generates oxygen vacancies, increasing the Co^3+^/Co^2+^ ratio. During the OER process, the octahedral Co─O active species in 4% Er‐Co_3_O_4_ are enhanced, indicating accelerated formation of key *O intermediates, which improve OER kinetics. This study demonstrates the successful manipulation of Co_3_O_4_ structure via metal doping for water electrolysis, offering valuable insights for the cost‐effective development of non‐precious metal electrocatalysts for acidic OER.

Tensile and compressive strain engineering have recently emerged as highly effective strategies for optimizing the performance of catalysts, particularly for the OER in acidic environments (Figure 5g).^[^ ^56^ ^]^ These approaches involve the deliberate introduction of mechanical strain at the atomic level to adjust the electronic structure and coordination environment of active metal sites, resulting in enhanced catalytic activity and stability. In tensile strain engineering, the expansion of lattice parameters leads to changes in bond lengths and atomic coordination. These structural modifications directly impact the electronic environment of the active sites, optimizing the binding energies of key reaction intermediates and improving the adsorption‐desorption kinetics crucial for OER.^[^ ^57^ ^]^ Mechanistically, tensile strain can lower the energy barriers for critical steps in the OER pathway, such as the adsorption of OH^−^ and the formation of oxygen‐containing intermediates, particularly OOH^*^.^[^ ^58^ ^]^ By shifting the d‐band center of transition metal catalysts, tensile strain fine‐tunes the adsorption energy of intermediates, thereby lowering the overpotential and improving intrinsic catalytic activity.^[^ ^59^ ^]^ This effect is especially valuable in acidic media, where catalysts often face increased dissolution rates and reduced stability.^[^ ^60^ ^]^ In addition to tensile strain, compressive strain also plays a significant role in catalysis. Yao et al. demonstrated that compressive strain in a Pt‐rich core‐shell structure significantly altered the electronic structure and redox behavior of Ru atoms anchored at specific sites (Figure 5h).^[^ ^61^ ^]^ By applying compressive strain, they optimized the binding of oxygen intermediates and improved resistance to over‐oxidation, enhancing the durability and activity of the Pt_3_Cu/Pt skin core‐shell catalysts. This highlights the potential of compressive strain to stabilize single‐atomic sites and extend catalyst lifetimes, a crucial factor in the development of robust catalysts for acidic OER applications. Similarly, the tensile strain applied to metal–organic frameworks (MOFs), as shown by the cobalt terephthalic acid (CoBDC) catalyst modified with ferrocene carboxylic acid (FcCA), modulated the spin state of Co active sites from high‐spin to intermediate‐spin.^[^ ^57^ ^]^ This adjustment optimized OH* adsorption energy, leading to efficient O─O bond formation and enhanced solar‐driven water splitting when paired with a BiVO_4_ photoanode. The findings underscore how tensile strain can regulate spin states and the electronic environment, overcoming common limitations of non‐carbonized MOF catalysts like poor conductivity and structural instability. Strain engineering also addresses the long‐standing issue of catalyst degradation in acidic environments. Xu et al. tailored the Ru─O bond covalency in Ru─Sn oxide matrices by introducing tensile strain, which prevented lattice oxygen participation and Ru dissolution, significantly improving the long‐term stability of the catalyst.^[^ ^29^ ^]^ This tensile strain also optimized the adsorption energy of intermediates, further boosting OER activity. The work highlights how tensile strain not only improves catalytic kinetics but also provides solutions to stability issues that limit the practical application of catalysts in harsh environments.

Grain boundaries can be introduced into crystalline materials during their growth through mechanisms such as stacking fault movements or oriented attachment. The presence of grain boundaries can enhance the catalytic activity of metals, as they provide flexibility in introducing and controlling compressive or tensile strain, which can be further optimized to improve catalytic performance. Additionally, due to the reversible nature of grain boundary structures, catalytic stability can also be maintained. Hao et al. innovatively developed a novel torsional strain Ta_0.1_Tm_0.1_Ir_0.8_O_2‐δ_ nanocatalyst, which exhibited outstanding activity and stability for acidic OER due to the synergistic effects of strain at the grain boundaries and doping (Figure 5i).^[^ ^62^ ^]^ The grain boundary‐induced strain primarily adjusted the Ir─O bond length, optimizing the adsorption energy of oxygen intermediates at the active Ir sites and lowering the energy barrier for OER, while maintaining excellent catalytic stability. Furthermore, the co‐doping of transition metal Ta and rare earth element Tm into IrO_2‐δ_ modified the electronic structure of the active sites, enhancing the binding energy of catalytic intermediates. Under industrial operating conditions, this novel torsional strain Ta_0.1_Tm_0.1_Ir_0.8_O_2‐δ_ nanocatalyst was successfully applied in PEM electrolyzers, achieving stable hydrogen production through water electrolysis. This study not only demonstrates an efficient OER nanocatalyst for promoting the industrial application of PEM electrolyzers but also introduces a new strategy for improving catalytic activity in electrochemical and other catalytic reactions using torsional strain nanostructured catalysts. In summary, strain engineering, through the application of both tensile and compressive strains, offers a powerful and versatile method to enhance the performance of OER catalysts. By precisely tuning the atomic and electronic structures of catalytic materials, strain engineering optimizes intermediate adsorption energies, modulates spin states, and enhances structural stability. This approach is particularly crucial for the development of next‐generation electrocatalysts capable of operating efficiently and stably in acidic media.

Innovative strategies also extend to low‐iridium content catalysts, which capitalize on their diverse elemental and structural compositions.^[^ ^63^ ^]^ These catalysts often involve the introduction of other elements or supports into iridium oxide matrices with a remained catalytic activity source of iridium oxide. For instance, perovskite‐type catalysts, characterized by their 3D crystal structures and flexible electronic configurations, exemplify the potential to design catalysts with simultaneous high activity and superior stability. By harnessing synergistic effects between different metal elements, it becomes feasible to achieve optimal orbital filling states and finely tune electronic structures, thereby creating an ideal bonding environment conducive to catalytic performance. Recent advancements have demonstrated that manipulating the interaction strengths between metal‐oxygen bonds can effectively address the dissolution challenges of constituent elements, as exemplified by the honeycomb layered structure of SrIr_2_O_6_.^[^ ^64^ ^]^ Because of its ultrashort Sr─O bonds and strengthened connectivity of IrO_6_ octahedral units, which is possible to effectively mitigate dissolution issues of constituent elements like Sr and Ir, thereby achieving exceptional structural stability under acidic OER conditions. There is increasing research awareness that many catalyst surfaces can undergo structural reconstruction in catalysis for OER in acid.^[^ ^9a^ ^]^ Similarly, Ruddlesden‐Popp phases like Sr_2_IrO_4_ exhibit a layered structure that not only exposes catalytic sites effectively but also maintains structural integrity through inherent ion exchange mechanisms, such as H^+^ and Sr^2+^ interactions, and hydrogen bonding between layers.^[^ ^65^ ^]^ Likewise, constructing metastable phase materials can bring promising catalytic properties due to their unique electronic environments, atomic arrangements, and crystal structures (Figure 5j).^[^ ^66^ ^]^ Zhou et al. report the phase‐selective synthesis of a metastable, open‐framework strontium iridate (γ‐SIO) for acidic OER.^[^ ^67^ ^]^ The γ‐SIO can be fully protonated in acid, further reconstitute ultra‐small, surfacing hydroxylated, (200) crystal‐oriented rutile nano catalyzers by protonation of iridium instead of the common amorphous IrO_x_H_y_ phase, a microstructure feature found to favor oxidation of hydroxyl groups and O─O bond formation in the electrocatalytic cycle. Theoretical calculations confirm that the (200) surface has more Ir active sites and lower overpotentials. Based on the above understanding of the structure‐activity‐stability relationship, we infer that reconstructing the electronic structure to alleviate dissolution during electrochemical processes is an effective method for enhancing stability. Improving the dispersion and intrinsic activity of IrO_x_ species remains a general strategy for constructing highly efficient electrocatalysts.^[^ ^68^ ^]^ In many cases, electrochemical surface reconstruction can generate highly dispersed Ir‐O^(II‐δ)−^‐Ir active species, which improve OER activity.^[^ ^69^ ^]^ For example, iridates like perovskites, pyrochlores, and weberite structures undergo electrochemical surface reconstruction to enhance their catalytic performance.^[^ ^70^ ^]^ Moreover, charge compensation using valence‐variable metals can effectively prevent excessive loss of lattice oxygen and reduce Ir dissolution during catalysis. By introducing additional surface proton acceptors, researchers have optimized the deprotonation pathway for OER intermediates, accelerating reaction kinetics. However, this approach can also risk covering pristine active sites. An innovative example of structural manipulation is the work by Chen et al., who developed high‐valence Mo‐modulated Pr_3_Ir_1‐x_Mo_x_O_7_ (xMo‐PIO), which triggers directional surface reconstruction by activating lattice oxygen and cations (Figure 5k).^[^ ^71^ ^]^ The activation of lattice oxygen and the self‐termination of Ir‐O_bri_‐Mo active species resulted in improved durability, as the Mo oxidation state compensated for Pr dissolution, effectively mitigating the loss of lattice oxygen.

### 3.2 Catalyst Macrostructure Regulation

The macrostructure of catalysts plays a pivotal role in determining their activity, stability, and overall performance in electrochemical reactions. In these systems, where the efficiency of hydrogen production relies heavily on the catalytic processes at both the anode and cathode, understanding how macrostructural factors influence performance is essential. Effective regulation of the catalyst macrostructure can be achieved through three key approaches: crystalline/amorphous construction, compositional engineering, and morphology control. Each of these strategies has a distinct mechanism through which it impacts catalyst activity and stability.

**Figure 6:**
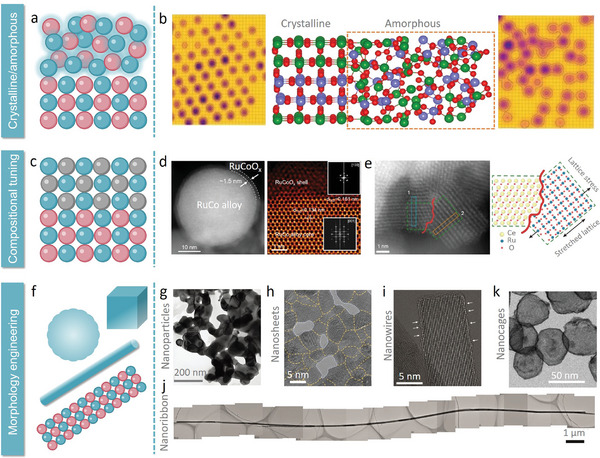
a) Schematic diagram of crystalline/amorphous construction. b) The structure of crystalline/amorphous MnRuO_x_ heterostructures with Gaussian fitting of STEM images. Reproduced with permission.^[^ ^73^ ^]^ Copyright 2024, Wiley. c) Schematic diagram of compositional tuning. d) High‐angle annular dark‐field scanning transmission electron microscope (HAADF‐STEM) image of the core/shell RuCo/RuCoO_x_ nanospheres and the heterojunction interface. Reproduced with permission.^[^ ^76^ ^]^ Copyright 2024, American Chemical Society. e) Aberration‐corrected HAADF‐STEM image of heterostructured RuO_2_‐CeO_2_ electrocatalyst. Reproduced with permission.^[^ ^77^ ^]^ Copyright 2024, American Chemical Society. f) Schematic diagram of morphology engineering. g) HRTEM image of py‐RuO_2_/Zn nanorods. Reproduced with permission.^[^ ^79^ ^]^ Copyright 2024, Wiley. h) HRTEM image of grain‐boundary‐rich porous RuO_2_ nanosheets. Reproduced with permission.^[^ ^81^ ^]^ Copyright 2024, Wiley. i) TEM image of RuFe nanoparticles. Reproduced with permission.^[^ ^82^ ^]^ Copyright 2023, Springer Nature. j) TEM image of Ir_3_Ni nanocages. Reproduced with permission.^[^ ^83^ ^]^ Copyright 2023, Springer Nature. k) TEM image of IrO_2_ nanowires. Reproduced with permission.^[^ ^85^ ^]^ Copyright 2024, American Chemical Society.

Phase engineering has gained attention, particularly the synthesis of amorphous/crystalline heterophase nanomaterials, which exhibit unique catalytic properties (**Figure** **6**a). The amorphous phase, with its randomly arranged and loosely bonded atoms, introduces a large number of unsaturated coordination sites and defects, significantly increasing the number of active sites.^[^ ^72^ ^]^ Amorphous catalysts are unique catalytic materials that combine short‐range order with long‐range disorder. While they resemble small‐scale cluster structures, amorphous catalysts exhibit random arrangements at larger scales, featuring isotropic and uniformly distributed active sites. Their high reactivity stems from distinctive coordination and a high concentration of unsaturated sites, which generate more active sites. Unsaturated bonds and elevated surface energy further enhance the OER activity. However, the limited specific surface area and inherent instability of amorphous catalysts restrict their stability and practical applications. Therefore, it is essential to explore the development of microcrystalline‐amorphous nanostructured materials to achieve a balance between activity and stability in electrocatalysis. Zhang et al. developed a simple Pechini method to synthesize the challenging MnRuO_x_ catalyst for acidic OER, where RuO_2_ microcrystalline regions serve as a supporting framework, and amorphous MnRuO_x_ fills the microcrystalline gaps (Figure 6b).^[^ ^73^ ^]^ The optimized amorphous/crystalline heterogeneous structure provides a large number of defects and active sites, facilitating the efficient adsorption and conversion of OH⁻ ions. Moreover, this heterogeneous structure elevates the d‐band center, bringing it closer to the Fermi level, thereby accelerating electron transfer and reducing charge transfer resistance at the active interfaces between the crystalline and amorphous phases. Moreover, the structural flexibility of the amorphous region allows it to adapt to and withstand structural disturbances during electrocatalysis, leading to improved stability for long‐term operations. For instance, amorphous/crystalline heterophase rutile‐structured RuO_2_ with Na doping and oxygen vacancies has demonstrated remarkable resistance to acid corrosion, Ru dissolution, and oxidation.^[^ ^74^ ^]^ Density functional theory (DFT) calculations revealed that Na doping and oxygen vacancies lower the binding energy of oxygenated intermediates on the RuO_2_ surface, thereby decreasing the activation energy required for OER.

Another effective approach involves coating catalyst surfaces with protective layers or films to act as barriers against corrosive media (Figure 6c). For instance, atomic layer deposition (ALD) of oxide or nitride thin films provides chemically inert surface layers that shield active sites from direct contact with acidic electrolytes, thereby enhancing catalyst material stability. Among them, Jiang et al. cleverly covered part of the RuO_2_ (110) crystal face with nitrogen‐doped carbon‐coated carbon nanotubes (NC/CNTs). The induced partial oxygen vacancies through C─O interactions, enhance the electronic coupling between NC/CNTs and RuO_2_ NPs.^[^ ^75^ ^]^ The strong electronic coupling between the NC/CNTs and the RuO_2_ increases the oxidation state and catalytic activity of Ru at the active site and improves the stabilities of lattice oxygen and surface Ru during the OER processes. Additionally, Cao et al. proposed a core/shell RuCo/RuCoO_x_ electrocatalyst, which features an enclosed Schottky heterojunction and an ultrathin conformal depletion layer (Figure 6d).^[^ ^76^ ^]^ This design maximizes the exposure of active sites in the depletion layer while optimizing the electronic structure, thereby enhancing the intrinsic activity and durability of the catalyst for acidic OER. The lattice strain and charge transfer induced by the Schottky heterojunction significantly affect the electronic structure of the active sites, modulating the OER pathway and suppressing the over‐oxidation and undesirable leaching of Ru species. Moreover, the enclosed core/shell structure effectively protects the metallic RuCo core from acidic corrosion, ensuring the integrity of the Schottky heterojunction during prolonged acidic OER testing. These advantages result in Ru‐based electrocatalysts demonstrating unprecedented durability and exceptional catalytic activity in acidic OER. In contrast to the core/shell heterostructure design, optimizing the lattice of materials to construct lattice‐matched/mismatched heterostructures is also an effective strategy. The degree of lattice matching at heterointerfaces influences the bonding between materials: well‐matched lattices create smooth interfaces conducive to charge carrier transport, while mismatched lattices may cause distortion and defects, impeding carrier mobility. Lu et al. constructed RuO_2_ and CeO_2_ nanoparticle heterostructures (Figure 6e).^[^ ^77^ ^]^ During the OER process, both interfacial and non‐interfacial sites of RuO_2_‐CeO_2_ followed the OPM and AEM mechanisms, respectively. At non‐interfacial RuO_2_ sites, an enhanced adsorption‐evolution mechanism (AEM‐plus) was observed, where lattice strain caused by lattice matching deformed the RuO_2_ structure and activated deprotonation of *OH near Ru active sites, promoting stable *OH adsorption and reducing the energy barrier of the AEM pathway. In contrast, the oxidation pathway mechanism (OPM) occurred at the RuO_2_‐CeO_2_ interface, where electron transfer between Ru and Ce atoms via a Ru─O─Ce bridge created a strong electronic coupling effect, directly connecting adjacent oxygen radicals. This lowered the overall reaction energy barrier and accelerated reaction kinetics. Furthermore, the Ru─O─Ce bridge inhibited Ru dissolution, contributing to the excellent stability of the RuO_2_‐CeO_2_ electrocatalyst under acidic conditions.

Morphological design also plays a critical role in the structural engineering of catalysts (Figure 6f). Constructing metastable nanostructures with distinct unit linkages can lead to entirely different active surfaces, providing unique catalytic properties.^[^ ^78^ ^]^ Ru‐based catalysts, particularly RuO_2_, exhibit high OER activity due to the optimal binding strength of OER intermediates (O*, OH*, and OOH*) at Ru sites. However, the over‐oxidation of Ru cations under acidic OER conditions can produce soluble species (Ru^n+^, n > 4), leading to rapid deactivation and significant performance loss. To enhance the OER performance of RuO_2_, strategies such as single‐atom guest doping (e.g., Ni, Pt) and lattice doping (e.g., Mn, Cu, Na) have been employed to modulate the chemical environment around Ru centers. Charge transfer between the guest atoms and Ru cations can modify the electronic structure of the Ru active sites. Introducing electron‐donating dopants into RuO_2_ reduces the oxidation state of Ru (Ru^n+^, n < 4), thereby protecting surface Ru cations from over‐oxidation to soluble species during the OER process. Based on this approach, Chen et al. synthesized Ru─Fe nanoparticles using an ultrafast Joule heating method, which demonstrated high OER activity and stability (Figure 6g).^[^ ^79^ ^]^ Under OER conditions, the surface structure of Ru─Fe nanoparticles transitions to an oxidized phase. This transformation not only enhances OER activity but also significantly improves the catalyst's stability in acidic environments by preventing the dissolution of active components and the over‐oxidation of Ru species. The introduction of Fe fine‐tunes the electronic structure of the catalyst, thereby reducing the energy barrier of the rate‐determining step (RDS).

Low‐dimensional materials, characterized by their anisotropic properties, quantum confinement effects, and edge phenomena, have become promising substrates for developing advanced catalysts.^[^ ^80^ ^]^ The increased surface energies of these materials further enhance their catalytic performance. Wang et al. proposed a grain boundary engineering strategy by developing ultrathin, porous RuO_2_ nanosheets with abundant grain boundaries as highly efficient acidic OER catalysts (Figure 6h).^[^ ^81^ ^]^ The presence of grain boundaries induced significant tensile strain, weakening the Ru─O covalency and shifting the Ru d‐band center upward, which enhanced the adsorption of oxygen intermediates and improved OER activity. The unsaturated coordination environment optimized intermediate adsorption, while the presence of grain boundaries effectively prevented the over‐oxidation of Ru, thereby stabilizing the structure of the active sites during the OER process. Furthermore, Lu et al. successfully synthesized a Zn‐doped RuO_2_ nanowire array catalyst (Figure 6i).^[^ ^82^ ^]^ The substitutional doping of Zn not only modulated the catalyst morphology but also generated abundant oxygen vacancies (V_O_) and low‐valence Ru sites. These V_O_ defects and Zn dopants effectively weakened the binding of oxygen adsorbates on the active Ru centers while enabling moderate *OH adsorption on Zn sites. As a result, the OER mechanism shifted from the traditional AEM pathway to a Ru─Zn dual‐site OPM mechanism, significantly enhancing OER activity. Additionally, the OPM mechanism prevented the over‐oxidation of metal sites, thereby protecting the active centers, while the Zn dopants and V_O_ defects helped maintain the structural stability of RuO_2_.

2D materials and 1D nanowires have shown significant promise in OER applications. It is important to note, however, that nanoribbons offer many advantages similar to, or even surpassing, those of 2D nanosheets, such as high surface area, enhanced charge transport, and favorable geometry for in situ detection. With rational design and manufacturing techniques, nanoribbon structures can be optimized for Ir‐based catalytic materials. Thermodynamically stable IrO_2_ typically exists in a rutile phase, where [IrO_6_] units are connected via shared edges or corners. Early studies suggest that the intrinsic activity of IrO_2_ is closely related to the connection of [IrO_6_] octahedral units and lattice distortions. Therefore, designing metastable nanostructures with different unit connections can provide entirely different active surfaces for electrocatalysis and offer deeper insights into structure‐activity relationships. Low‐dimensional materials with increased surface energy, due to their inherent anisotropy, quantum confinement effects, and edge states, offer a platform for developing advanced catalysts. Shao et al. constructed ultralong nanowires with a metastable nanostructure (Figure 6j), identified as a monoclinic phase with a C2/m (12) space group, evolving from monoclinic K_0.25_IrO_2_.^[^ ^47e^ ^]^ In addition to exposing more active edge sites, the Ir atoms in monoclinic IrO_2_ nanorods (NRs) possess lower d‐band energy levels compared to rutile IrO_2_, leading to weaker adsorption of *O intermediates. This self‐regulating four‐electron OER process enables balanced free energy distribution, resulting in a lower overpotential.^[^ ^83^ ^]^

The rise of sub‐nanometer structures has introduced a new dimension to the design of OER electrocatalysts, offering extensive possibilities for optimizing active sites. Wu et al. successfully developed sub‐nanoporous Ir_3_Ni ultrathin nanocages with high crystallinity (Figure 6k).^[^ ^84^ ^]^ The sub‐nanoporous shell facilitates the optimal exposure of Ir_3_Ni nanocage active sites. Ni doping was shown to reduce the RDS energy barrier. Notably, the Ir_3_Ni ultrathin nanocages undergo surface reconstruction during OER catalysis, forming a nickel‐incorporated Ir‐rich oxide outer layer. This structural transformation plays a pivotal role in enhancing both OER activity and stability. Furthermore, the high crystallinity of the nanostructure ensures robustness, providing sustained catalytic performance during the oxygen evolution reaction, even after surface oxidation. As surface science, nanoscience, and nanotechnology rapidly advance, along with theoretical studies, the development of well‐defined metal nanostructures and a deeper understanding of their structure‐activity relationships will pave the way for the next generation of OER electrocatalysts.

### 3.3 Support Optimization

The support material is a necessary condition for anchoring the active sites, thereby preventing the dissolution of active sites due to strong metal‐support interactions. Selecting a proper support material is crucial to fully utilize the stabilizing effect of the support, as it must be both resistant to corrosion and electronically tunable at the same time.^[^ ^85^ ^]^ The evolution of substrate materials significantly impacts OER efficiency. Graphene and its derivatives have emerged as promising candidates for OER substrates due to their excellent conductivity and large surface area.^[^ ^86^ ^]^ Their 2D structure not only provides ample anchoring sites for active species but also enhances charge transfer kinetics, thereby improving catalytic activity. Additionally, graphene‐based materials exhibit notable chemical stability under acidic environments, ensuring sustained performance over extended operational cycles. In contrast, traditional carbon supports like Vulcan XC‐72 carbon black, although widely used, show limited surface functionalization and poor corrosion resistance at low pH.^[^ ^87^ ^]^ This underscores the need for substrates capable of withstanding harsh electrochemical conditions while maintaining structural integrity.

Theoretically, certain transition metal oxides (Sn/Mn/W/Ti/Nb‐based oxides) exhibit potential as suitable substrates.^[^ ^88^ ^]^ Currently, TiO_2_ substrates are widely utilized; however, due to their inherent poor electrical conductivity, significant efforts have been dedicated to optimizing them through morphology construction and chemical modification. Similarly, Nb_2_O_5_ and ZrO_2_ are emerging as focal points in current research.^[^ ^89^ ^]^ It is crucial to recognize that the dispersion pattern (atoms or layers covered on the support) significantly impacts both its activity and longevity.^[^ ^48^, ^90^ ^]^ The bonding and distribution between the catalyst and substrate are influenced by structural compatibility.^[^ ^48a^ ^]^ Structural compatibility refers to having the same crystal type or matched crystal planes between the catalyst and substrate, facilitating the formation of a stable and homogeneous composite structure. For instance, in the case of IrO_2_, the lattice structure of TiO_2_ closely resembles that of IrO_2_. Such structural similarity enhances the stability and activity of the catalyst by providing a secure foundation for the dispersion and immobilization of IrO_2_ nanoparticles. Therefore, when selecting a substrate for catalyst hosting, considerations must encompass its conductivity, chemical and oxidative stability, morphology, dispersal capabilities, surface charging properties, as well as its thermodynamic and kinetic characteristics. These factors are crucial for ensuring the stability of the OER in acidic environments.

When selecting the optimal substrate for acidic OER, researchers must also consider the interactions between the substrate and active sites. For example, certain oxides, initially challenging to stabilize theoretically, can serve as stable substrates through structural reconfigurations, giving rise to novel reaction mechanisms facilitated by this unique catalyst structure. Lee et al. have reported an electrocatalyst with Ru‐atom‐array patches supported on α‐MnO_2_ (Ru/MnO_2_) for the oxygen evolution reaction following a mechanism that involves only *O and *OH species as intermediates.^[^ ^91^ ^]^ This mechanism allows direct O─O radical coupling for O_2_ evolution, which is named oxygen pathway mechanism (OPM).^[^ ^91^ ^]^ The positions of the Ru atoms follow the periodic arrangement of the Mn sites in the crystalline α‐MnO_2_, resulting in the formation of small, regularly arranged Ru ensembles. The cation exchange reaction that occurred in situ during the OER triggers the reconstruction of the small Ru ensembles into large patches of Ru atom arrays. The changes of the metal concentration in the electrolyte and the electrocatalyst structure were tracked to further understand the OER process via ICP‐OES. This evolution of the substrate‐catalyst structure enhances OER efficiency while ensuring the stability of both the substrate and the catalytically active sites. Furthermore, Liu et. al. report a chromium‐iridium oxide catalyst with high performance to address the above challenges. The catalyst has a strong coupling between active IrO_2_ and corrosion‐resistant CrO_2_, which can optimize the intermediate adsorption energy for OER and strengthen the Ir─O bond, hence endowing it with high activity and durability.^[^ ^92^ ^]^ In addition, transition metal oxides (e.g., TiO_2_, SnO_2_), known for their oxygen affinity, tend to form strong bonds with active species, thereby enhancing durability but potentially altering electronic properties crucial for efficient OER kinetics.^[^ ^85b^ ^]^ However, transition metal oxides suffer from poor conductivity, necessitating either higher catalyst loading or modifications to improve electrical conductivity. While metal nitrides exhibit good conductivity, they gradually oxidize and phase transition into oxynitrides and oxides under acidic conditions, compromising their excellent initial electrical properties. Therefore, there is a strong motivation to develop new support materials that balance long‐term stability with sufficient electronic conductivity. Cho et al. synthesized Zr_2_ON_2_ as a carrier material for IrO_2_, leveraging the excellent conductivity of ZrN and stability of ZrO_2_, thus achieving a balance between acidic OER activity and stability.^[^ ^93^ ^]^

**Figure 7:**
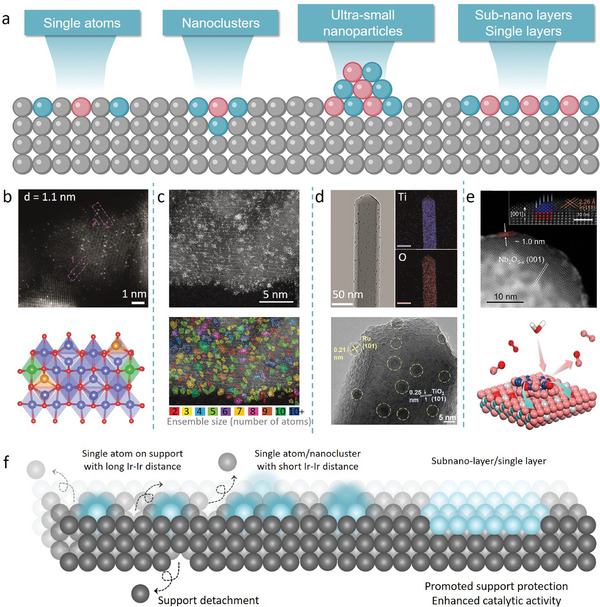
a) Schematic structure of the catalyst‐support interface. b) HAADF‐STEM image of Ir_1_/Cu_0.3_Co_2.7_O_4_. Reproduced with permission.^[^ ^95^ ^]^ Copyright 2024, Springer Nature. (c) Aberration corrected the HAADF‐STEM image of Ir single‐atom‐ensembles (Ir_SAE_) stabilized on the surface of Mn‐substituted spinel Co_3_O_4_ and the corresponding Ir_SAE_ size distribution graph. Reproduced with permission.^[^ ^96^ ^]^ Copyright 2024, Wiley. d) TEM and HRTEM images of Ru/TiO_x_. Reproduced with permission.^[^ ^97^ ^]^ Copyright 2023, Springer Nature. e) HAADF‐STEM image of Ir/Nb_2_O_5‐x_ and corresponding dynamic interface structure. Reproduced with permission.^[^ ^98^ ^]^ Copyright 2022, Wiley. f) Schematic diagram of optimization strategy for single‐atomic catalytic materials on support.

The optimization of active sites can enhance the stability of the supporting materials, thereby further improving the overall activity and durability of the catalyst (**Figure** **7**a). In the case of transition metal oxide supports, the weak interaction between single atoms and the support, as well as the lack of strong atomic spatial correlation (i.e., the arrangement of neighboring single atoms on the oxide support), makes the single‐atom bonding highly susceptible to corrosion or dissolution, especially under harsh acidic OER conditions, leading to significant deactivation. Therefore, effective strategies are urgently needed to manipulate the coordination environment of active single atoms and optimize their short‐range spatial correlation by precisely adjusting the surface energy of oxide supports. Zeng et al. introduced acid‐resistant Ir single atoms into the lattice of spinel cobalt oxide, which significantly suppressed Co dissolution and maintained high stability during acidic OER (Figure 7b).^[^ ^94^ ^]^ The incorporation of Ir single atoms notably increased the migration energy of neighboring Co atoms while having minimal effect on more distant Co atoms. As the Ir–Ir distance decreased to a limit of 0.56 nm, where Co atoms were sandwiched between two Ir atoms, the electrochemical durability of the catalyst in acidic conditions progressively improved, and Co dissolution was substantially reduced. Considering the excessive participation of lattice oxygen in transition metal oxide supports, which leads to metal leaching and structural collapse, hindering their practical application, Ashwani et al. proposed a simple strategy to regulate the surface energy of the spinel Co_3_O_4_ support through Mn modification (Figure 7c).^[^ ^95^ ^]^ They synthesized Ir single‐atom catalysts stabilized on Mn‐substituted spinel Co_3_O_4_, effectively catalyzing slow acidic OER in harsh oxidative environments. Compared to pristine Co_3_O_4_, Mn substitution at the octahedral sites significantly altered the surface binding energy of the Co_3_O_4_ support, reducing the migration energy barrier of isolated Ir single atoms (Ir_SA_) on the surface. This promoted the formation of strongly correlated Ir single‐atom ensembles (Ir_SAEs_) during pyrolysis. The rigid Ir_SAEs_ stabilized at the material surface with an appropriate Ir–Ir distance, effectively suppressed lattice oxygen participation while promoting direct O─O radical coupling, mitigating Ir dissolution and structural collapse, and enhancing stability in acidic environments.

A similar strategy can be applied to the design and optimization of catalyst materials based on stable supports. Lv et al. developed Ru nanoparticles supported on non‐stoichiometric TiO_x_ nanorods with atomic‐level defects, serving as effective acidic OER electrocatalysts (Figure 7d).^[^ ^96^ ^]^ A spontaneous redox reaction between Ru ions and the Ti substrate introduced oxygen vacancy defects at the edges of TiO_2_ nanorods. The introduction of these oxygen vacancies facilitated the stable adsorption of Ru^5+^ on the TiO_x_ support, enhanced the oxidation resistance of the Ru/TiO_x_ system, and lowered the energy barrier of the rate‐determining step in the OER. Moreover, the strong interaction between Ru and the TiO_x_ substrate stabilized the valence state of the Ru active centers, further improving OER stability. Similarly, Xing et al. used Ir supported on Nb_2_O_5‐x_ (Ir/ Nb_2_O_5‐x_) as a model catalyst and identified that bias‐induced dynamic oxygen species migration at the catalyst/support interface significantly promoted OER performance (Figure 7e).^[^ ^97^ ^]^ Under different applied biases, oxygen species migrated from Nb_2_O_5‐x_ to the Ir nanoparticles, generating an Ir─O coordination structure, while excess oxygen on the Ir surface transferred back to Nb_2_O_5‐x_ via Nb^4+^. This dynamic migration of oxygen species at the interface not only introduced a new IrO_x_ variable in the OER process, breaking traditional scaling relationships, but also maintained the Ir centers in a lower oxidation state at challenging high potentials, ensuring the stability of IrO_x_.

Considering recent advancements, the future direction of catalyst design can be predicted (Figure 7f). For single‐atom catalysts, the limited interaction between single atoms and the support, as well as the lack of atomic spatial correlation, hinders the synergy between Ir–Ir active sites, resulting in limited activity enhancement. Additionally, the exposed support materials are prone to dissolution under harsh OER conditions. While the direct design of atomic clusters or ultra‐fine nanocrystals can enhance activity to some extent, it inevitably reduces atomic efficiency. To balance these advantages, future design goals could focus on sub‐nano‐layer catalysts or even single‐layer catalysts. This approach would not only ensure effective linking between Ir–Ir active sites and improve atomic utilization but also provide reverse protection by stabilizing the catalytic layer and supporting material.

### 3.4 Noble Metal‐Free Catalysts Design

PEMWEs have traditionally relied on noble metal catalysts, particularly iridium‐based oxides, to achieve the high activity and stability required for the OER in acidic environments. While these catalysts demonstrate exceptional performance, their widespread adoption is hindered by the high cost, scarcity, and limited annual production of iridium (≈8 metric tons/year). At current iridium loadings of 2 ≈ 4 mg cm^−2^, the global capacity of PEMWEs is restricted to ≈10 GW year^−1^, far from meeting the demands of large‐scale hydrogen production.^[^ ^4c^ ^]^ These limitations have spurred significant research into noble metal‐free catalysts as a more abundant and cost‐effective alternative. Although noble metal‐free catalysts, including transition metal oxides, nitrides, and single‐atom catalysts, have demonstrated certain acidic OER activity via advanced structural engineering and defect modulation,^[^ ^88^, ^98^ ^]^ their limited stability still restricts further applications. Thus, enhancing the structural and electrochemical stability of noble metal‐free catalysts is a crucial step toward overcoming these challenges and unlocking the sustainable hydrogen production potential of PEMWEs.

Low‐cost transition metals (e.g., cobalt) and their oxides are known to be active for OER in alkaline electrolytes. However, their performance in acidic electrolytes remains limited. Thus, designing an OER catalyst with excellent activity and durability in acidic electrolytes is of great significance. Science reported a nanofibrous cobalt spinel catalyst co‐doped with lanthanum and manganese, which was prepared from zeolitic imidazolate frameworks embedded in electrospun polymer fibers.^[^ ^99^ ^]^ The approach selectively incorporates oversized, stable secondary elements onto the cobalt oxide surface to induce strain, oxygen vacancies, and acid resistance, thereby enhancing OER activity in acidic media. Incorporating a third element with a similar charge and size to cobalt into the lattice interior can bridge the Fermi bandgap through partial occupation of the d‐orbitals caused by d‐electron delocalization, thus improving the electrical conductivity. The catalyst's high porosity and large surface area facilitate effective reactant accessibility, while the electrode layer ensures efficient H_2_O transport and O_2_ release. Furthermore, the metal oxide catalyst demonstrates stability against oxidation and acidic corrosion in PEMWE. This catalyst exhibits a low overpotential of 353 mV at 10 mA cm^−2^ and minimal OER activity degradation over 360 h in acidic electrolytes. When incorporated as the anode catalyst in PEMWE, it delivers a current density of 2000 mA cm^−2^ at 2.47 V (with a Nafion 115 membrane) or 3.00 V (with a Nafion 212 membrane) and exhibits low degradation rates during accelerated stress tests.

A fundamental understanding of the OER mechanisms involving single‐ and dual‐nuclear reaction intermediates and catalytic pathways is essential for guiding the design of precursors and catalysts with lower overpotentials and enhanced acid resistance. Following this design principle, an amorphous Mo─Ce oxide‐supported single‐atom Co catalyst, encapsulated within bamboo‐like carbon nanotubes, exhibits remarkable OER performance in acidic media.^[^ ^100^ ^]^ Operando studies reveal that Mo─Ce oxide stabilizes the Co‐active sites, facilitates the LOM pathway, reduces overpotential, and enhances durability. The confinement effect of carbon nanotubes minimizes corrosion, achieving long‐term stability exceeding 60 h in PEMWEs at high current densities. Similarly, defect engineering has been employed to improve the activity and stability of spinel cobalt oxides.^[^ ^101^ ^]^ Vacancy‐rich Co_3_O_4_ hollow nanocubes (V_O_‐Co_3_O_4_ HNCs) display excellent OER activity and stability, with an overpotential of 265 mV at 10 mA cm^−2^ and the ability to operate for 130 h under acidic conditions at 20 mA cm^−2^. The introduction of oxygen vacancies optimizes the adsorption/desorption energy barriers of reaction intermediates, suppressing Co dissolution via the LOM pathway. These structural modifications, combined with the hollow structure, increase the exposure of active sites, enable high current densities, and render V_O_‐Co_3_O_4_ as a viable candidate for industrial‐scale PEM applications.

Despite the ongoing challenges in achieving catalytic activity and stability comparable to noble metal catalysts under harsh acidic conditions, the current work offers forward‐looking directions and design insights for the future development of noble‐metal‐free OER catalysts for hydrogen production via PEMWE technology. For instance, increasing the density of surface functional groups through element doping, primary size control, and morphological innovation can further boost catalytic activity. Optimizing the electronic structure via computational modeling and tailoring the interface between the catalyst and the support, along with removing electrochemically unconnected oxides, can enhance the intrinsic activity and prevent degradation, thus improving material durability. Future efforts should focus on scalable synthesis, operando characterization to understand degradation mechanisms, and the innovative design of catalysts that balance cost, activity, and durability. By addressing these challenges, non‐noble metal catalysts can play a pivotal role in transforming PEMWEs into a cost‐effective and sustainable hydrogen production method.

## 4 Methods to Improve Stability of MEA

When translating the insights from mechanistic studies into practical electrolyzer systems, it is crucial to understand that the performance and stability of these systems will vary based on different reaction environments. Effective optimization of proton, electron, and gas transfer interfaces is essential for ensuring stable operation, minimizing maintenance needs, and controlling operating costs.^[^ ^102^ ^]^ Given the critical role that these interfaces play in the overall efficiency and longevity of PEMWEs, addressing the factors affecting their performance is paramount. In particular, the interfaces between the PEM/CL interface, and the CL/PTL interface, require meticulous optimization.^[^ ^5^, ^12^ ^]^ These interfaces are central to the efficient transfer of protons, electrons, and gases, and their performance directly impacts the stability of the PEMWEs. At the same time, understanding these dynamics is crucial for optimizing PTL design and enhancing the performance and longevity of PEMWE systems in practical applications. For instance, Danilovic et al. conducted further research on the impact of PTL and their interfaces in PEMWEs. The bulk properties of PTL have a substantial influence on mass transport resistance within PEMWEs by affecting the distribution and movement of water and oxygen across these layers. Their study utilized a lattice Boltzmann method (LBM) in conjunction with PTL tomography to investigate oxygen transport dynamics in PTLs. As depicted in the study, the structure of PTLs significantly influences oxygen distribution.^[^ ^103^ ^]^ PTLs with high porosity and low tortuosity facilitate efficient oxygen removal and enhance water permeability, thereby reducing mass transport resistance during PEMWE operation. Optimal PTL microstructures promote effective utilization of catalyst layers and facilitate two‐phase flow, thereby enhancing overall performance and potentially extending durability. Operating in a regime where mass transport is limited can compromise long‐term stability. To bridge the gap between optimal PTL microstructure and enhanced catalyst utilization, it is crucial to analyze the conduction mechanisms within anodic catalyst layers. In addition, in a gaseous water environment, ionomer thin film phases in catalyst layers experience moderate swelling, which minimally affects the electrical percolation network of catalyst agglomerates.^[^ ^104^ ^]^ However, at ionomer loadings exceeding 3 wt%, swelling in liquid water induces mechanical distortion of this network, resulting in highly tortuous electron pathways due to disconnected agglomerates.^[^ ^105^ ^]^ Consequently, the three‐phase boundaries at active catalyst sites cannot be sustained, rendering disconnected catalyst layer domains electrochemically inactive. The microporous layer offers an advantage through localized compression of the catalyst layer at the microscopic level, which helps maintain the integrity of the electrical catalyst percolation network, thus preventing disruption.

This section focuses on elucidating the fundamental concepts and specific methods for enhancing these interfaces. By improving the integration and interaction between the PEM/CL interface, as well as the CL/PTL interface, it is possible to achieve more effective proton and electron transport, better gas distribution, and reduced degradation. Addressing these aspects involves various strategies such as advanced interface engineering, innovative material design, and optimized coating techniques. These measures aim to enhance the durability and efficiency of PEMWEs, ensuring robust and reliable operation in practical applications.

### 4.1 PEM/CL Interface Engineering

The engineering of the PEM/CL interface is crucial for optimizing the performance and stability of PEMWEs. This interface plays a key role in enhancing proton conductivity, which is essential for efficient electrochemical reactions and high electrolyzer performance. Effective interface engineering ensures consistent catalyst activity by providing uniform proton contact, reducing ohmic losses through minimized resistive barriers, and preventing degradation by maintaining robust contact between the PEM and CL. Therefore, by minimizing resistive barriers and ensuring stable contact, well‐designed interfaces contribute to improved overall system stability.

#### 4.1.1 Direct Membrane Deposition

**Figure 8:**
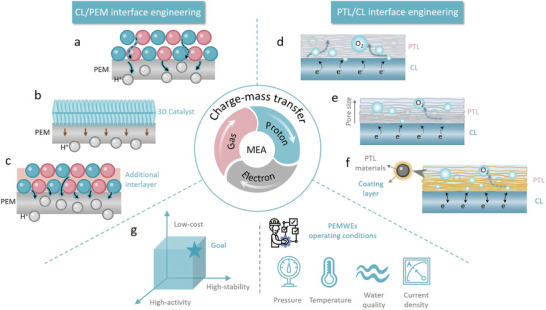
a) Schematic illustrations of the CL/PEM interface and proton transport. b) 3D structural design of catalyst layer/proton exchange membrane. c) Additional interlayer. d) Schematic illustrations of the PTL/CL interface and oxygen/electron transport. e) Gradient pore design of PTL. f) PTL coating layer. g) Relative operating parameters of practical PEMWE devices.

One effective approach to optimizing the PEM and CL interface is through direct membrane deposition (**Figure** **8**a). This method involves applying the PEM material directly onto the catalyst layer, thereby establishing a close and intimate contact between these two critical components.^[^ ^106^ ^]^ By eliminating intermediate gaps or barriers, direct deposition enhances proton conductivity by facilitating a more direct and efficient pathway for proton transfer from the membrane to the catalyst. This technique also reduces interface resistance, which is crucial for minimizing energy losses and improving overall electrolyzer efficiency. Moreover, direct deposition promotes a uniform and continuous interface, which is essential for consistent proton transport across the entire active area of the catalyst. This uniformity ensures that the entire catalyst surface receives a steady supply of protons, which helps maintain optimal catalytic activity and performance. Holzapfel et al. developed PEMWE‐MEAs using direct membrane deposition (DMD). This method involved spray coating the membrane directly onto the substrate. Polarization experiments with DMD‐MEAs revealed promising results, demonstrating superior electrochemical performance compared to reference catalyst‐coated membranes (CCMs) and porous transport electrode (PTE) MEAs with freestanding Nafion 117 membranes.^[^ ^107^ ^]^ The DMD approach offers a streamlined fabrication process and greater design flexibility for PEMWE‐MEA manufacturing through its simple layer‐by‐layer technique. The improved adhesion and integration between the PEM and CL achieved through direct membrane deposition not only enhances the efficiency of electrochemical reactions but also contributes to the stability and longevity of the membrane electrode assembly, ultimately leading to more reliable and effective operation of the proton exchange membrane water electrolyzer.

#### 4.1.2 3D Interface

3D interface engineering represents an advanced method for optimizing the PEM/CL interface (Figure 8b).^[^ ^108^ ^]^ This approach involves creating a 3D structured interface that promotes better integration and contact between the PEM and CL. By incorporating features such as porous or patterned structures, the 3D interface design can enhance proton transmission pathways and increase the effective contact area. This not only improves the proton conductivity but also supports the distribution of reactants and removal of products, contributing to more stable and efficient electrolyzer operation. For instance, Tian et al. created an ordered 3D conical Nafion array on a commercial Nafion 115 membrane using anodic aluminum oxide (AAO) template and deposited the Ir catalyst onto this 3D structure via direct sputtering.^[^ ^109^ ^]^ This approach significantly enhanced catalyst utilization achieved excellent low‐Ir catalytic performance, and resulted in a MEA with reduced ohmic resistance. Similarly, Dong et al. developed a novel nanoimprinting technique to fabricate an anode with a gradient 3D conical array (GTA) in PEM water electrolysis.^[^ ^110^ ^]^ This method formed a robust 3D interface with an expanded surface area, leading to improved contact and transport at the PEM/catalyst layer interface. Compared to a conventional electrolyzer with the same Ir loading, the MEA featuring the GTA demonstrated superior performance due to these enhancements.

#### 4.1.3 Additional Layer

Another strategy is the incorporation of an additional intermediate layer between the PEM and the CL (Figure 8c). This intermediate layer is specifically engineered to enhance proton conductivity and improve adhesion between the two surfaces. Typically, as the Nafion layer penetrates into CL, creating a rougher and more extensive interface that facilitates better proton transport. This layer is designed to possess high ionic conductivity and compatibility with both the PEM and CL materials, effectively bridging any gaps or inconsistencies at the interface. As a result, overall proton transport efficiency is improved, and the likelihood of performance degradation over time is reduced. Furthermore, the Nafion interlayer provides significant mechanical stability; it acts as a robust binder between the CL and PEM, thereby preventing delamination during stability tests. Additionally, Yang et al. have referred to this additional layer as a “highway” for protons and electrons.^[^ ^111^ ^]^ By incorporating a conductive phase, such as gold, into the intermediate layer, rapid electron transport between the PEM and the catalyst layer is achieved, further enhancing the electrochemical performance of the system.

### 4.2 PTL/CL Interface Engineering

The interface between the CL and the PTL is crucial for optimizing both electronic transmission and gas bubble management in PEMWEs (Figure 8d). The CL must effectively transfer electrons to the PTL, which in turn conducts these electrons to the external circuit. This requires a high‐quality electrical contact to minimize resistance and ensure efficient electron flow. In addition, oxygen gas bubbles generated at the anode must be efficiently detached and removed during electrolysis. The PTL must offer high electrical conductivity and appropriate porosity to support both effective electron transfer and gas management. To achieve optimal performance, engineers focused on improving interface contact through surface treatments and patterned the PTL to balance effective gas removal with minimal electrical resistance and maintain the corrosion resistance of the substrate.

#### 4.2.1 PTL Patterned Design

Implementing a patterned design for the PTL helps optimize gas distribution and flow within the electrochemical cell (Figure 8e). By engineering specific patterns, such as flow fields or microchannels, the PTL can ensure more uniform reactant distribution and efficient removal of reaction byproducts. This reduces localized pressure build‐up and prevents issues such as gas crossover or bubble formation that could otherwise disrupt the catalytic reactions and degrade performance. Additionally, well‐designed PTL patterns can enhance the overall mechanical stability of the cell by distributing stresses more evenly, thereby reducing the likelihood of mechanical failure.

#### 4.2.2 PTL Coating Layer

Coating a catalyst layer onto the PTL is pivotal for optimizing the performance and durability of PEMWEs (Figure 8f). The catalyst layer provides a protective shield that improves corrosion resistance, shielding the PTL from the harsh electrolytic environment and prolonging the lifespan of the system. It also mitigates passivation resistance by reducing the formation of passivation layers that can otherwise hinder catalytic activity. Importantly, a well‐loaded catalyst layer like Pt helps minimize hydrogen crossover into the oxygen stream, thus reducing the risk of safety hazards and performance issues associated with hydrogen contamination. Overall, the strategic coating of the catalyst layer on the PTL is essential for achieving enhanced efficiency, durability, and safety in PEMWE systems.

### 4.3 Operating Conditions Optimization

Beyond the mechanistic studies discussed earlier, it is evident that the deactivation mechanisms of PEMWEs are intimately connected to their operating conditions. When scaling up from lab‐scale testing to commercial‐scale electrolyzers, a thorough consideration of these operating conditions is essential. These conditions include electrolytes, impurities, and various operational parameters such as temperature, pressure, current density, local pH, and bubble formation, all of which play crucial roles in the design and functionality of practical PEMWE systems for sustained operation (Figure 8g).

Temperature, while necessary for optimizing reaction kinetics, can also accelerate the degradation of membrane and catalyst materials if not properly controlled. Excessive temperatures can lead to thermal stress, reducing material durability and overall system reliability. Pressure affects the solubility and diffusion rates of gases within the electrolyzer. Elevated pressures can enhance reaction rates but may also cause mechanical strain on the cell components, potentially leading to physical failures or reduced operational lifespan.

Current density, a critical factor in determining the electrolyzer's performance, influences the efficiency of the electrochemical reactions. However, excessively high current densities can result in increased ohmic losses, higher temperatures, and accelerated wear of the membrane and catalyst materials. Local pH variations can significantly impact the stability and performance of the proton exchange membrane and catalysts. Deviations from optimal pH levels can lead to increased corrosion rates, membrane degradation, and loss of catalytic activity.

The presence of impurities, such as metal ions or other contaminants, can severely affect the performance of PEMWEs. Impurities can cause metal poisoning, leading to reduced catalytic efficiency and increased ohmic resistance. Additionally, bubble formation during the electrochemical reactions can disrupt the uniform distribution of reactants and byproducts, leading to localized pressure fluctuations, reduced reaction efficiency, and potential mechanical damage to the cell components. In summary, understanding and controlling these operating parameters is critical for optimizing the performance, durability, and efficiency of PEMWE systems. Effective management of temperature, pressure, current density, local pH, and purity is essential to prevent degradation, ensure stable operation, and enhance the long‐term reliability of commercial‐scale PEMWEs.

### 4.4 Membrane Electrode Optimization

The optimization of membrane electrodes is also crucial to improving the overall catalytic efficiency of PEMWEs, alleviating gas crossover, and improving device safety. It is mainly divided into two main strategies: optimization of the proton exchange membrane and adjustment of the combination of the proton exchange membrane and the catalyst.

#### 4.4.1 Optimization of the Proton Exchange Membrane

**Figure 9:**
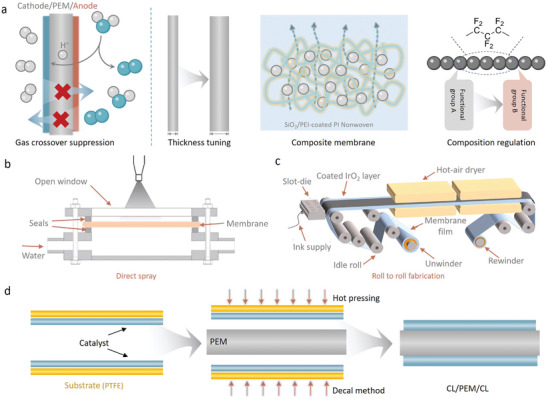
a) Strategies to mitigate gas crossover. Increased thickness, composite membranes, and modified membrane chemical composition. Schematic diagram of different membrane electrode preparation methods, b) direct‐spray method, Reproduced with permission.^[^ ^116^ ^]^ Copyright 2017, Elsevier. c) roll‐to‐roll coating process, Reproduced with permission.^[^ ^117^ ^]^ Copyright 2020, Elsevier. and d) decal transfer process.

The optimization of the PEM involves several strategies, including regulating thickness, designing composite membranes, and advancing polymer components (**Figure** **9**a). Thicker membranes can better withstand deformation, tearing, or delamination under operational stress, which is essential for maintaining long‐term performance. However, while increasing thickness can enhance mechanical stability, it must be balanced against the potential rise in ohmic resistance, which could negatively impact electrochemical performance. Therefore, careful optimization is necessary to ensure that the advantages of increased thickness do not compromise conductivity.

In addition to thickness regulation, the development of proton exchange membrane materials is crucial. As summarized in **Table** **1**, the main types of proton exchange membranes, including perfluorinated, partially fluorinated, hydrocarbon‐based, composite, and polybenzimidazole (PBI‐based) membranes, exhibit distinct advantages and limitations.^[^ ^112^ ^]^ For example, perfluorinated membranes like Nafion dominate due to their exceptional proton conductivity and chemical stability but come with high costs and environmental concerns. Innovations in polymer chemistry, particularly the creation of new fluorinated and non‐fluorinated polymers, can significantly improve proton conductivity while reducing costs. In contrast, hydrocarbon‐based membranes such as sulfonated polyether ether ketone (SPEEK) offer cost‐effectiveness and environmental benefits but require improvements in durability and conductivity for widespread adoption.^[^ ^113^ ^]^ Furthermore, incorporating ion‐conductive fillers, such as sulfonated nanoparticles, into the membrane matrix can enhance both proton conductivity and mechanical stability. These composite structures can enhance overall thickness while preserving proton conductivity. Moreover, the added layers can serve as barriers to gas diffusion, effectively facilitating proton transport.

**Table 1:** Comparison of PEM types.

Type	Proton conductivity	Thermal stability	Durability	Cost	Examples	Application focus
Perfluorinated	High	Moderate (≈80 °C)	Excellent	High	Nafion (DuPont) Aquivion (Solvay)	PEMFC PEMWE
Partially Fluorinated	Moderate	Moderate (≈80 °C)	Moderate	Moderate	Fumapem F‐950	PEMFC
Hydrocarbon‐Based	Moderate	Moderate (≈120 °C)	Moderate	Low	SPEEK, SPPS, Sulfonated polyimides	PEMFC
PBI‐Based	Low (low temp.) High (high temp.)	High (≈200 °C)	High	High	PBI‐H_3_PO_4_ Celtec (BASF)	High‐temperature PEMFC
Composite Membranes	High (tunable)	High (≈150 °C)	High	Moderate	SPEEK + silica Nafion + zirconia PBI + ZrO_2_	PEMWE, high‐temperature PEMFC

Overall, the optimization of proton exchange membranes is a multifaceted process that focuses on improving proton conductivity, mechanical stability, and chemical durability through various strategies. These optimizations are vital for enhancing the performance and longevity of proton exchange membrane fuel cells (PEMFCs) and PEMWEs, ultimately contributing to their commercial viability and efficiency in sustainable energy applications.

#### 4.4.2 Catalyst Coating Techniques

Catalyst coating technology plays a key role in improving interfacial properties.^[^ ^114^ ^]^ Selecting the appropriate catalyst type and coating method plays a pivotal role in optimizing both activity and durability. **Table** **2** summarizes the classifications, advantages, and limitations of noble metal‐based catalysts, non‐precious metal catalysts, and hybrid catalysts. Noble metal catalysts exhibit unparalleled activity and stability in acidic environments but are hindered by high costs and limited availability. Noble metal‐free catalysts, such as transition metal oxides or nitrides, offer a cost‐effective alternative with promising activity through defect engineering and structural optimization, though stability remains a challenge. Hybrid catalysts attempt to balance cost and performance, leveraging the synergy between precious and non‐precious materials.

**Table 2:** Comparative summary of advantages and disadvantages of different catalysts.

Category		Examples	Advantages	Disadvantages
Noble Metal Catalysts	Noble Metal Oxides	IrO_2_ RuO_2_	High activity and stability High electrical conductivity Well‐studied	High cost Scarcity of iridium Limited availability
	Mixed Metal Oxides	Ir_x_Ru_1‐x_O_2_ SrIrO_3_ Ti‐doped IrO_2_	Synergistic effects improve catalytic performance Reduced noble metal usage	Still relies on noble metals Challenge in optimize composition
	Single‐Atom Catalysts	Ir/Ru SACs	High atom utilization efficiency Tunable catalytic properties	Challenging synthesis Stability issues in harsh conditions
Noble Metal‐free Catalysts	Transition Metal Oxides	Co_3_O_4_ MnO_x_ NiFe_2_O_4_	Cost‐effective and abundant Moderate activity in alkaline media	Poor stability in acidic media Susceptible to corrosion under acidic conditions
	Transition Metal Nitrides/ Phosphides/Sulfides	CoN, NiN, FeN CoP, FeP, NiP CoS_x_, NiS_x_	Good conductivity Intermediate catalytic performance	Oxidation in acidic media Requires surface protection
	Perovskite Oxides	La_0.5_Sr_0.5_CoO_3_ Ba_0.5_Sr_0.5_Co_0.8_Fe_0.2_O_3_	High structural flexibility Tunable electronic structure	Limited stability in acidic conditions Complex synthesis
	MOF‐Derived Catalysts	Co‐MOF‐derived Co/Co_3_O_4_ Fe‐MOF‐derived Fe/Fe_3_O_4_	High surface area Tunable pore structure Abundant active sites	Complex preparation Limited durability in acidic conditions
	Carbon‐Based Composites	Co‐embedded in N‐doped carbon graphene‐supported catalysts	High conductivity Synergistic effects with carbon matrix	Corrosion in acidic conditions Limited long‐term stability
	Single‐Atom Catalysts	Co SACs	High atom utilization efficiency Tunable catalytic properties	Challenging synthesis Stability under harsh conditions

The choice of coating technique must align with the specific catalyst type to maximize its potential. For instance:

#### 4.4.3 Direct Spray

Direct Spray Coating is a technique where catalyst materials are applied directly onto the electrode surface using a spray mechanism (Figure 9b).^[^ ^115^ ^]^ The direct spray process comprises two main stages: ink preparation and application. The ink consists of a blend of catalyst, ionomer, and an appropriate solvent, which may include isopropyl alcohol, ethanol, water, or more commonly, a combination of these solvents. Following uniform dispersion of the ink through ultrasonic treatment, it is applied to the stationary PEM using either an air spray gun or ultrasonic spray equipment. Finally, it is dried at high temperature. This method provides precise control over catalyst loading and allows for the deposition of various catalyst materials. And it is suitable for high‐performance noble metal catalysts in both small‐scale and customized applications. Direct spray coating offers flexibility in catalyst formulation and is relatively straightforward to implement. However, achieving consistent coating thickness can be challenging, particularly for hybrid catalysts requiring intricate structural control, and the technique may require careful optimization of spray parameters to avoid issues like uneven deposition, excessive material waste, and solvent effect on PEM.

#### 4.4.4 Roll to Roll Fabrication

Roll‐to‐Roll Fabrication is a continuous process designed for large‐scale production (Figure 9c), this method supports NPMCs and hybrid catalysts.^[^ ^116^ ^]^ In this technique, catalyst materials are deposited onto a moving substrate, such as a PEM, using methods like slot‐die coating or gravure printing. This method ensures uniform catalyst distribution over extensive areas and is well‐suited for high‐volume manufacturing. And ideal for producing electrodes in bulk, particularly where cost‐efficiency and scalability are critical. Roll‐to‐roll fabrication allows for consistent coverage and is economically advantageous for large‐scale production. But maintaining consistent adhesion and dispersion over long production runs can be challenging, and the technique may involve complex setup and calibration to ensure optimal performance.

#### 4.4.5 Decal Method

The Decal Method involves transferring a pre‐coated catalyst layer from a temporary carrier to the final electrode substrate. In this method, an ink comprising an ionomer, catalyst, and solvent is applied to a substrate, typically a PTFE or Kapton film (Figure 9d). This technique excels in achieving smooth interfaces and controlled catalyst loadings, which are essential for optimizing the performance of noble and hybrid catalysts. The PEM is positioned between two coated substrates and subjected to hot pressing at a controlled pressure and temperature for a specified duration. Following the hot‐pressing process, the substrates are peeled away, leaving behind two thin layers of catalyst on the PEM, thereby creating a catalyst‐coated membrane (CCM). This method offers high precision in controlling the thickness and uniformity of the catalyst layer, making it particularly suitable for intricate catalyst patterns and high‐resolution coatings. Its application, however, is less suitable for certain NPMCs that demand high‐temperature annealing or in situ structural adjustments. And the process is more labor‐intensive and involves additional steps, which can increase production time and complexity.

Each coating technique offers distinct benefits and limitations, and the selection of an appropriate method depends on factors such as production scale, cost considerations, and the specific requirements of the PEMWE application. The integration of catalyst classification with tailored coating methods can bridge the gap between laboratory‐scale development and industrial‐scale production. Future advancements should focus on refining coating techniques to maintain catalyst integrity while improving interfacial properties, enabling cost‐effective and durable PEM device applications.

## 5 Outlooks and Challenges

The enduring stability of acidic OER is crucial for enabling widespread commercial deployment of PEMWEs, yet overcoming this remains a significant grand challenge. Continued research efforts are warranted to address these complexities and pave the way for practical advancements in PEMWE technology. Based on the mechanistic insights and challenges outlined in this review, several future perspectives and strategies can be proposed for the development of catalysts with enhanced stability. Further investigations are essential to understand the interactions between catalysts and electrolyzer components, advancing toward the goal of achieving sustainable long‐term electrolysis.

### 5.1 Degradation Mechanism Investigation

Components of the electrolyzer, including membranes, interface resistance, flow fields, and gas diffusion layers, significantly influence the overall durability of industrial water electrolysis.^[^ ^43^ ^]^ Therefore, we advocate for a deeper study and characterization of the main components within the electrolyzer to investigate the degradation mechanism, including theoretical calculations as well as in situ characterization. These analyses can yield crucial insights into the stable catalytic structures, offering valuable information for advancing the field.

#### 5.1.1 Separating Anode Overpotential from the PEMWEs

**Figure 10:**
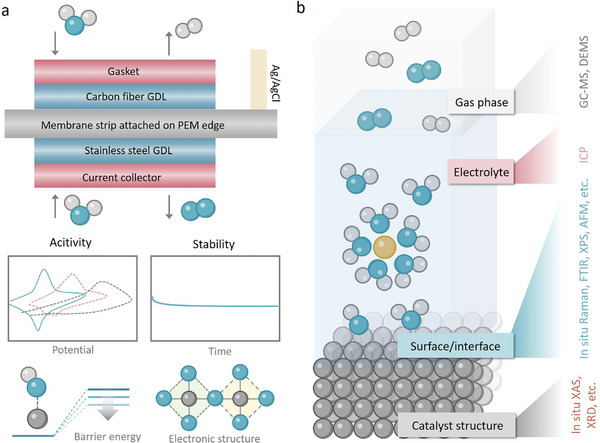
a) Schematic showing the integration of the reference electrode in the AEMWE system for PEM performance and corresponding theoretical analysis for mechanism investigation. b) Schematic of in situ characterizations based on above the improved AEMWE system.

In PEMWE systems, isolating the anode overpotential is crucial for enhancing performance. Current dual‐electrode measurement methods often fail to thoroughly investigate the underlying causes of performance variations in PEMWE, particularly when adjusting specific parameters or comparing different interfacial engineering approaches. Conventional MEA configurations only record the total cell voltage, and the absence of liquid electrolytes complicates the integration of reference electrodes. Reference electrodes are widely used in flow battery setups to measure half‐cell potentials, but their application in PEMWE is limited. To reduce operational costs and improve energy efficiency, accurately analyzing the sources of overpotential is essential. Such analysis aids researchers in identifying components or interfaces that require improvement and in understanding how changes in individual parameters affect the performance of each unit. Recent advancements include the development of novel diagnostic systems designed to independently assess overpotentials associated with various components and their interfaces. An innovative diagnostic approach involves connecting a membrane strip, functioning as an ion conductor, to the PEM outside the active region and linking it to an Ag/AgCl reference electrode within the PEM water electrolyzer (**Figure** **10**a). This setup allows for independent measurement of the potentials at the anode and cathode, underscoring the importance of developing innovative diagnostic tools for PEMWE devices. This method not only enhances the accuracy of performance assessments but also provides valuable data to support subsequent optimization and improvements, making PEMWE systems more effective and widely applicable in the field of renewable energy conversion and storage. Future research may focus on optimizing this diagnostic system for real‐time monitoring and adjustment, thereby enhancing the overall performance of PEMWE. Through more detailed potential analyses, we can gain deeper insights into the interactions of various components, ultimately advancing the application of PEMWE technology in sustainable hydrogen production.

#### 5.1.2 In Situ Characterization Techniques

Understanding the degradation mechanisms of proton exchange membrane systems (PEMWEs and PEMFCs) is critical for enhancing their durability and performance. Current in situ characterization techniques often rely on three‐electrode systems,^[^ ^117^ ^]^ which provide valuable insights into catalytic behavior but fall short of replicating the complex operational conditions of PEM devices. These simplified setups fail to capture the intricate interactions and dynamic changes occurring at the CL, PTLs, and MEA under real‐world conditions such as fluctuating humidity, temperature, and high current densities. To overcome these limitations, operando characterization strategies that integrate advanced techniques with operational PEM environments have emerged as a promising direction.

In situ characterization offers invaluable experimental data and profound insights into reaction mechanisms, essential for optimizing catalyst design, refining electrolyzer structure, enhancing operational conditions, and boosting energy conversion efficiency (Figure 10b).^[^ ^118^ ^]^ For instance, in situ electrochemical techniques like EIS and CV enable real‐time monitoring of kinetic behaviors and interfacial properties such as catalyst activity, proton transport rates, and kinetics of oxygen redox and hydroxide reactions. Leveraging these techniques allows for precise optimization of electrolyzer design to enhance both efficiency and stability. Synchrotron‐based X‐ray absorption spectroscopy (XAS) enables real‐time monitoring of changes in the electronic structure and oxidation states of catalyst active sites during the OER or HER.^[^ ^119^ ^]^ This technique is particularly effective for studying LOM pathways and identifying degradation‐related phenomena, such as active site dissolution and phase transformations. Additionally, in situ X‐ray absorption fine structure (XAFS) technology can detect real‐time changes in the bulk‐phase structure of catalytic materials, providing insights into the true reaction states of catalysts during the electrolysis process.^[^ ^120^ ^]^ Spectroscopic methods such as in situ infrared (IR) and Raman spectroscopy provide crucial information on reaction intermediates and surface products.^[^ ^121^ ^]^ This knowledge aids in understanding electrochemical reaction mechanisms and optimizing catalyst performance and operational parameters. Additionally, environmental transmission electron microscopy (TEM) allows the visualization of structural changes in catalysts and MEAs under conditions mimicking the operational environment. For example, it can track the evolution of catalyst nanoparticles, the formation of interfacial voids, and the aggregation or dissolution of active species, which are key factors in performance degradation. Coupling these tools with spectroscopic techniques such as Raman or infrared spectroscopy can provide complementary insights into chemical bonding and intermediate species on the catalyst surface.

The design of operando‐compatible characterization cells tailored to PEM devices is crucial. Such cells must accommodate the unique requirements of PEM systems, including proton conductivity, high humidity, and operational pressures while maintaining compatibility with advanced characterization equipment. The design of multifunctional electrodes requires the effective integration of photoelectrodes, electrochemical sensors, and spectroscopic detectors to achieve comprehensive real‐time monitoring. For example, microfluidic platforms and transparent cells integrated with synchrotron light sources have been successfully used to probe dynamic processes in PEM systems under realistic conditions. This design allows researchers to capture data at different stages of the electrolyzer operation, enabling a more thorough analysis of system behavior. Furthermore, intelligent electrolysis electrolyzer systems, integrating advanced sensing technologies and automated control systems, enable real‐time monitoring and intelligent adjustment of operational conditions. This approach enhances the stability, controllability, and longevity of electrolyzers, reduces energy consumption, and supports the commercialization of PEMWEs and PEMFCs technology. The integration of these advanced operando techniques not only bridges the gap between laboratory‐scale studies and practical applications but also provides a deeper understanding of key degradation mechanisms. Insights gained from these studies can guide the rational design of more robust catalysts, membranes, and MEAs by identifying critical failure modes and optimizing material properties to mitigate degradation.

In summary, the application of in situ characterization techniques and innovative electrolyzer designs provide essential tools for understanding the complex electrochemical processes in PEMWEs and PEMFCs. This knowledge is indispensable for optimizing electrolyzers, enhancing energy conversion efficiency, and promoting the widespread adoption of this technology in clean energy applications. Future development should focus on continuously improving in situ characterization techniques to achieve greater precision and efficiency in the development of electrolyzers. The integration of advanced technologies, such as machine learning and data analysis, holds promise for a deeper understanding of electrolyzer behavior and more effective optimization. By leveraging these tools, researchers can establish predictive frameworks to design next‐generation PEM systems with enhanced durability and efficiency, paving the way for their widespread adoption in clean energy applications.

#### 5.1.3 Theoretical Calculations

Theoretical calculations provide valuable insights into catalyst deactivation mechanisms under acidic conditions, which can be simulated and validated using computational methods such as Density Functional Theory (DFT), COMSOL simulations, and machine learning.^[^ ^122^ ^]^ These approaches elucidate specific deactivation pathways and influential factors. For instance, DFT calculations enable the design of more durable catalysts by studying electronic structure, activation energy, and adsorption energies on catalyst surfaces. This predictive capability aids in screening catalysts for acidic OER environments, supporting the development of industrial‐grade stability standards. Theoretical simulations also play a crucial role in devising catalyst regeneration strategies. By analyzing the causes of deactivation, effective regeneration processes can be designed to restore catalyst activity, extending its operational lifespan and reducing production costs. COMSOL simulations are instrumental in modeling processes like electric field distribution, proton transport, and electrochemical reactions within electrolyzer systems. This helps optimize electrolyzer structures, enhance energy conversion efficiency, and deepen understanding of reaction mechanisms. Machine learning algorithms leverage vast DFT computational and experimental datasets to extract patterns and correlations. This capability aids in predicting the activity and stability of new materials, optimizing catalyst synthesis processes, and refining control strategies for electrolyzer systems to improve efficiency and stability. Totally, the application of theoretical computational simulations to understand catalyst deactivation in acidic OERs not only enhances comprehension of deactivation mechanisms and catalyst design optimization but also boosts the reliability and cost‐effectiveness of industrial applications. These advancements accelerate material development and improve proton exchange membrane water electrolysis cell technology, advancing water electrolysis for energy conversion and storage applications.

### 5.2 Industrial Applicable PEMWE Optimization

Industrial‐scale PEMWE systems face significant challenges in achieving high efficiency, durability, and cost‐effectiveness simultaneously. The target set by the U.S. DOE aims for 3 A cm^−2^ at a cell voltage of 1.9 V by 2050. This goal demands higher current densities and operating temperatures (60 to 80 °C), imposing stringent requirements on catalytic activity, mass/electron transfer efficiency, and overall device stability compared to lab‐scale tests conducted at lower current densities and room temperature. Current PEMWE technologies often struggle to maintain stable performance over extended operational periods due to catalyst degradation, membrane deterioration, and electrode fouling. Addressing these challenges requires a comprehensive optimization approach encompassing materials design, system integration, and operational strategies. Key advancements in industrial‐applicable PEMWE optimization include:

#### 5.2.1 Advanced Catalyst Design

Currently, commercially available electrochemical catalysts for acidic OER primarily utilize noble metals such as iridium and ruthenium due to their high activity and stability. To enhance catalyst durability and reduce costs, there is an ongoing exploration of using earth‐abundant elements, such as iron, nickel, manganese, and cobalt, as alternative materials, thereby reducing reliance on noble metals. The design of catalyst nanostructures incorporates stable support materials combined with single‐atom layers to improve catalytic efficiency while ensuring the overall stability of the materials. The design of these advanced catalysts emphasizes the suppression of degradation mechanisms, which is essential for maintaining catalytic performance during prolonged operation and establishing a foundation for the widespread application of PEMWE.

#### 5.2.2 Optimized PEM

The intrinsic physical properties of PEM directly impact proton conduction efficiency and the overall performance of electrolyzers. Current research focuses on improving PFSA polymers and the application of derived polymeric materials. These materials must not only exhibit excellent conductivity but also possess outstanding chemical stability and thermal resistance to withstand harsh operating conditions. Additionally, enhancing the interfacial compatibility between the PEM and catalysts through the functionalization of membrane materials is a key area of investigation. The development of novel membrane materials will facilitate the further maturation and application of PEMWE technology.

#### 5.2.3 Thermal Management and Electrode Stratification

High‐power PEMWE operation introduces substantial thermal stresses, which can lead to catalyst layer delamination, membrane expansion, and system inefficiencies. Effective thermal management strategies are therefore essential. These include designing heat‐resistant electrode structures and incorporating thermally conductive materials into the cell assembly to ensure uniform temperature distribution. Additionally, stratified electrode designs, where distinct layers optimize catalytic activity, mass transport, and structural stability, can enhance overall performance and durability.

#### 5.2.4 Gas‐Liquid Management

Efficient gas‐liquid management is crucial for maintaining reactant access and removing generated gases to prevent flooding or dry‐out in the cell. Optimized flow field designs, such as interdigitated or serpentine configurations, improve reactant distribution and gas removal. Employing advanced hydrophobic coatings and tailoring porosity in GDLs can further enhance water management and prevent performance losses under high current densities.

#### 5.2.5 System Engineering

The integration and optimization of advanced catalysts, optimized PEM, and electrode configurations are crucial in PEMWE systems. Systematic studies involve a comprehensive consideration of electrode structure, flow field design, and operational parameters such as temperature, pressure, and current density. Optimizing these parameters not only maximizes the energy conversion efficiency of the electrolyzer but also effectively reduces the rate of equipment aging. For example, implementing various flow field designs can improve the mass transport efficiency of reactants, thereby enhancing the performance of the electrolyzer. Additionally, dynamically adjusting operational parameters to accommodate changes in the operating state of the electrolyzer is an important strategy for improving overall efficiency.

#### 5.2.6 Durability and Lifetime Assessment

To ensure the long‐term operation of PEMWE systems, durability and lifespan assessments are essential. Implementing rigorous testing protocols, including accelerated aging studies and simulations of actual operating conditions, facilitates a comprehensive understanding of material degradation mechanisms. By developing predictive models aligned with experimental data, researchers can more effectively devise maintenance strategies and optimize system longevity. This process not only aids in evaluating the system's reliability but also provides crucial data support for practical industrial applications. With advancements in technology, the adoption of more sophisticated monitoring techniques, such as online sensor technology, has also emerged as a trend in current research.

#### 5.2.7 Cost Reduction Strategies

Reducing the cost of PEMWE is crucial for its commercialization. To achieve economically viable water electrolysis, multiple approaches can be explored, including the development of more cost‐effective materials and manufacturing processes. For instance, employing recycling strategies for key materials used in electrolysis not only lowers material costs but also minimizes resource waste. Additionally, implementing innovative production methods, such as 3D printing technology, can enhance manufacturing efficiency and reduce costs. Optimizing operational conditions, improving system design, and increasing overall energy efficiency are also essential components in lowering the costs associated with PEMWE technology. The implementation of these strategies will facilitate the commercialization of PEMWE technology and promote its application in the clean energy sector.

#### 5.2.8 Leveraging PEMFC Advancements

The durability challenges faced by PEMFCs, such as catalyst dissolution, carbon support corrosion, and membrane degradation, bear significant similarities to those encountered in PEMWE systems.^[^ ^123^ ^]^ For example, the use of alloyed noble metals, such as Pt alloys in PEMFCs, has demonstrated improved resistance to dissolution and corrosion, which can be adapted to iridium‐based catalysts in PEMWEs to enhance their long‐term stability. Similarly, the degradation mechanisms of PFSA membranes under oxidative and hydrolytic conditions in PEMFCs provide critical lessons for improving PEMs in electrolyzers. PEMFC flow‐field designs also offer direct applicability to PEMWE. Advanced designs that ensure even gas distribution and efficient water management have been instrumental in mitigating mass transport limitations at high current densities. For instance, interdigitated and serpentine flow fields optimized for gas reactant supply in PEMFCs can be adapted to improve the gas‐liquid separation and oxygen removal processes in PEMWEs, especially under high‐current‐density operation. These parallels underscore the importance of leveraging the extensive research on PEMFCs to enhance PEMWE system stability and efficiency.

#### 5.2.9 Cross‐Learning from AWE and AEMWE

Insights from alkaline water electrolyzers (AWEs) and anion exchange membrane water electrolyzers (AEMWEs) offer additional perspectives for optimizing PEMWE systems. In AWEs, the use of earth‐abundant and cost‐effective catalysts, such as Ni‐based materials, has enabled stable operations under alkaline conditions. This suggests opportunities for PEMWE to integrate stable electrode structures that combine the robustness of alkaline catalysts with the efficiency of acidic environments, such as hybrid catalyst designs. Additionally, the scalability of electrode configurations in AWEs provides a template for designing cost‐effective and durable electrode assemblies in PEMWEs. From AEMWEs, the development of ion‐exchange membranes with high hydroxide conductivity and chemical stability presents parallels to the challenges faced in PEMWE membrane optimization. For instance, AEMWE research has highlighted the importance of ionomer distribution within catalyst layers to enhance catalyst‐membrane interface compatibility, which can be adapted to improve proton transport efficiency in PEMWEs. Furthermore, the ongoing exploration of dual‐ion conducting membranes in AEMWEs suggests innovative pathways for enhancing the performance of PEMs in PEMWE systems. By synthesizing findings from these related technologies, PEMWE research can adopt a multidisciplinary approach to address its unique challenges, including reducing costs, improving stability, and scaling systems for industrial applications.

### 5.3 Unified Optimization Strategy

The advancement of PEMWE technology requires adopting a multidisciplinary approach that bridges knowledge from PEMFCs, AWEs, and AEMWEs. Leveraging advanced characterization techniques and modeling tools commonly used in PEMFCs can deepen our understanding of degradation mechanisms in PEMWEs. For instance, in situ spectroscopy methods used to monitor catalyst dissolution in PEMFCs can be employed to study similar processes in PEMWE under real‐time operational conditions. Collaborative research between fuel cell and electrolyzer communities is crucial for accelerating innovation. Developing unified computational models that integrate findings across these systems could predict performance trends, identify potential bottlenecks, and guide the design of next‐generation PEMWE systems. Such collaborations would facilitate cross‐pollination of ideas and technologies, leading to faster adoption of sustainable hydrogen production technologies. By learning from related fields and embracing a systems‐level optimization framework, PEMWE research can overcome existing limitations and realize its full potential for industrial‐scale applications.

To sum up, advancing industrial‐applicable PEMWE optimization demands interdisciplinary collaboration among material scientists, electrochemists, engineers, and industry stakeholders. By addressing fundamental challenges in catalyst design, membrane technology, system engineering, and cost‐efficiency, we can accelerate the adoption of sustainable hydrogen production technologies. Particularly, scaling up PEMWE systems to meet high‐power demands requires careful consideration of key factors such as thermal management, electrode stratification, and gas‐liquid management. Effective thermal management solutions are essential for maintaining system stability and preventing thermal‐induced degradation while optimizing electrode design and structure can enhance performance and durability under high current densities. Moreover, improving gas‐liquid management through advanced flow field designs and membrane innovations will ensure efficient reactant delivery and product removal, preventing flooding or drying out of the system. Continued research and development efforts will be pivotal in unlocking the full potential of PEMWE for industrial applications, driving toward a greener and more resilient energy landscape. Ultimately, by overcoming these challenges and optimizing the system design for high‐power operation, PEMWE can play a central role in large‐scale, sustainable hydrogen production, contributing to the transition to a low‐carbon economy and supporting the global shift toward clean energy solutions.

## Conflict of Interest

The authors declare no conflict of interest.
